# Unraveling the genetics of feline hypertrophic cardiomyopathy: a multiomics study of 138 cats

**DOI:** 10.1093/g3journal/jkaf153

**Published:** 2025-07-03

**Authors:** Joanna L Kaplan, Victor N Rivas, Michael W Vandewege, Jalena R Wouters, Samantha P Harris, Katherine M Meurs, Joshua A Stern

**Affiliations:** Department of Medicine and Epidemiology, School of Veterinary Medicine, University of California, Davis, Davis, CA 95616, United States; The Feline Health Center, College of Veterinary Medicine, North Carolina State University, Raleigh, NC 27606, United States; Department of Clinical Sciences, College of Veterinary Medicine, North Carolina State University, Raleigh, NC 27606, United States; Department of Clinical Sciences, College of Veterinary Medicine, North Carolina State University, Raleigh, NC 27606, United States; Department of Medicine and Epidemiology, School of Veterinary Medicine, University of California, Davis, Davis, CA 95616, United States; Department of Physiology, College of Medicine-Tucson, University of Arizona, Tucson, AZ 85724, United States; The Feline Health Center, College of Veterinary Medicine, North Carolina State University, Raleigh, NC 27606, United States; Department of Clinical Sciences, College of Veterinary Medicine, North Carolina State University, Raleigh, NC 27606, United States; The Feline Health Center, College of Veterinary Medicine, North Carolina State University, Raleigh, NC 27606, United States; Department of Clinical Sciences, College of Veterinary Medicine, North Carolina State University, Raleigh, NC 27606, United States

**Keywords:** whole-genome sequencing, RNA-sequencing, precision medicine, cardiovascular, translational

## Abstract

Hypertrophic cardiomyopathy (HCM) is the most common inherited cardiac disease in cats, often leading to congestive heart failure, arterial thromboembolism, and sudden cardiac death. The genetics of feline HCM are poorly understood and limited genetic discoveries remain breed- or family-specific. We aimed to identify novel causative or disease-modifying variants in a large cohort of cats reflective of the general cat population. In a second cohort, we sought to characterize transcriptomics differences between HCM-affected cats and healthy controls. DNA was isolated from 138 domestic cats (109 HCM and 29 controls). No single or combination of variants of high, moderate, or modifying impact were identified by genome-wide analysis to cause or modify disease severity of HCM. Several rare high and moderate impact variants in genes associated with human HCM were detected in diseased cats. In a second cohort, left ventricular (LV), interventricular septal (IVS), and left atrial (LA) tissues of 27 HCM-affected and 15 control cats were submitted for stranded mature RNA-sequencing at 50 million reads/sample. A total of 74, 115, and 45 differentially expressed genes (DEGs) were upregulated and 8, 53, and 48 DEGs were downregulated in LV posterior wall, IVS, and LA tissue, respectively, in HCM-affected cats compared to controls. Similar to humans, the genetic etiology of feline HCM remains unknown in a high proportion of cases. Transcriptomics revealed molecular signatures that may help identify novel HCM biomarkers or drug targets in future investigations.

## Teaser

Feline hypertrophic cardiomyopathy (HCM) is considered hereditary, although genetic discovery is limited to specific breeds or small families. Kaplan *et al*. performed whole genome sequencing and RNA-sequencing in 138 and 42 cats, respectively, to identify novel variants and molecular signatures associated with disease. No single or combination of variants appeared to cause or modify disease in whole genome association testing, highlighting the complex disease genotype-phenotype relationship. Transcriptomic data revealed several differences in gene expression profiles between HCM-affected cats and controls that may serve useful in future investigations of feline precision medicine.

## Introduction

Hypertrophic cardiomyopathy (HCM) afflicts 1 in 7 cats and frequently leads to life-limiting outcomes including left-sided congestive heart failure (CHF), arterial thromboembolism (ATE), hemodynamically significant arrhythmias, and sudden cardiac death (SCD) (Payne *et al.* 2015; Luis Fuentes *et al.* 2020; Kittleson and Cote 2021). The disease is defined as primary pathologic asymmetrical or symmetrical hypertrophy of the left ventricular posterior wall (LVPW) and interventricular septum (IVS) (Luis Fuentes *et al.* 2020; Kittleson and Cote 2021). A confirmatory antemortem diagnosis requires documentation of left ventricular wall thickening on echocardiogram and exclusion of all secondary causes of hypertrophy, including systemic hypertension, hyperthyroidism, hypersomatotropism, infiltrative inflammatory or neoplastic diseases, storage disorders, and outflow tract obstructions (ie subaortic stenosis).

Feline HCM is largely considered hereditary in origin, although only a small number of breed-specific genetic mutations are recognized in the veterinary literature (Meurs *et al.* 2005, 2007, 2021; Luis Fuentes *et al.* 2020; Kittleson and Cote 2021). The first causative variant was identified in the Maine Coon cat as a single nucleotide polymorphism (SNP) in codon 31 in the *MYBPC3* gene (MYBPC3:c91G>A[A31P]) (Meurs *et al.* 2005). This variant led to an amino acid change from alanine to proline in cardiac myosin binding protein C. Shortly after, a pathogenic single base pair substitution in codon 820 causing an amino acid change from arginine to tryptophan in *MYBPC3* was identified in Ragdoll breed cats (MYBPC3:c.2453C>T[R820W]) (Meurs *et al.* 2007). Although other variants have been reported ranging from unknown significance to likely pathogenic, only the MYBPC3:c.2453C>T[R820W] and the MYBPC3:c91G>A[A31P] variants reach pathogenic classification according to the American College of Medical Genetics and Genomics Guidelines (Schipper *et al.* 2019; McNamara *et al.* 2020; Meurs *et al.* 2021; Schipper *et al.* 2022; Boeykens *et al.* 2024). More recently, a nonsarcomeric variant in *ALMS1* (ALMS1:c.7384G>C[G2462R]) was reported in Sphynx cats and proposed as a cause of HCM in the breed (Meurs *et al.* 2021). However, alternate sample cohorts failed to replicate this finding (Boeykens *et al.* 2024). Additional studies identified variants within isolated families or individual cats affected with HCM, but failed to confirm a true association with disease (Schipper *et al.* 2019, 2022; McNamara *et al.* 2020). Identification of breed-specific genetic mutations has altered breeding and screening recommendations for Maine Coon and Ragdoll cats, resulting in a reduced incidence of HCM and earlier recognition of this disease in these breeds (Longeri *et al.* 2013). In addition, discovery of causative genetic mutations in feline HCM has aided in the discovery of causative genetic mutations in human HCM. For example, the discovery of the variant MYBPC3:c.2453C>T[R820W] in Ragdolls helped lead to the identification of this same mutation in an affected Italian family (Ripoll Vera *et al.* 2010). Finally, the presence of the naturally occurring MYBPC3:c91G>A[A31P] variant in a family of purpose-bred cats has allowed the domestic cat to serve as an invaluable translational model for discovery of drugs for human HCM such as mavacamten and aficamten (Stern *et al.* 2016; Sharpe, Oldach, Kaplan, *et al.* 2023; Sharpe, Oldach, Rivas, *et al.* 2023). Unfortunately, the genetics of HCM in the majority of domestic cats remain unknown (O'Donnell *et al.* 2021; Kaplan *et al.* 2023; Rivas *et al.* 2023). Furthermore, among affected cats with both known and unknown genetic etiologies, expression of disease phenotypes and clinical outcomes are highly heterogenous. Significant knowledge gaps in the genetic and molecular pathways involved in initiating and promoting the development of HCM not only limit the translational potential of the domestic cat with HCM but also make it challenging to effectively prognosticate clinical outcomes and develop more targeted screening, risk stratification, and management strategies for the individual patient.

In humans with HCM, enormous strides in genetic discovery since the 1990s have led to the identification of over 1,500 mutations in >11 genes considered causative or associated with disease (Maron *et al.* 2012; Freeman *et al.* 2017; de Marvao *et al.* 2021; Writing Committee Members *et al.* 2021; Haraf *et al.* 2024). Of those with an identified genetic mutation, approximately 70% lie within either the *MYH7* or *MYBPC3* gene. Investigations that begin to elucidate the genetic and molecular makeup of disease have allowed for enhanced screening programs and rapid development of novel drug therapies (McNamara *et al.* 2015, 2016; Green *et al.* 2016; Stern *et al.* 2016; Schmid and Toepfer 2021; Haraf *et al.* 2024). Yet, in approximately 30% of human cases, the underlying genetic cause remains undetermined (Maron *et al.* 2012; Freeman *et al.* 2017). Similar to cats, identifying a single causative mutation in humans does not necessarily predict disease outcome, as phenotypic expression of disease is highly variable even among those with shared genetic mutations (Freeman *et al.* 2017). However, a recent polygenic approach suggested that common genetic variants occurring in a compound fashion were linked with HCM in human patients without a previously identified genetic mutation (Harper *et al.* 2021). Furthermore, in patients with a known sarcomeric mutation, the presence of multiple pathogenic variants led to more severe disease. Certain disease-associated mutations are not only exonic, but also found in intronic or regulatory regions (Lopez Castel *et al.* 2010; Mitina *et al.* 2024). For example, Friedreich’s ataxia is a neurodegenerative disease associated with HCM (Al-Mahdawi *et al.* 2008; Weidemann *et al.* 2012). A homozygous GAA repeat expansion mutation within intron 1 of the *FXN* gene is suggested to promote both genomic and epigenetic changes that lead to the development of concurrent HCM. This combination of findings suggests that multiple mechanisms may influence disease modification and outcome. We suspect that similar mechanisms occur in cats and aim to further explore the genetics of HCM in the broader feline population.

Given observations in humans, we hypothesize that genetic variants across sarcomeric and ion-channel genes are the etiologic drivers of feline HCM. Our aim was to characterize genetic and gene expression differences between HCM-affected cats with varying disease severity and clinical outcomes. Therefore, we explored whole-genome sequencing (WGS) data for disease variants that explain a high proportion of feline HCM and characterize genetic variation in genes commonly linked to HCM. We additionally summarized RNA expression differences between healthy and HCM-affected heart tissues.

## Methods

*Ethics approval*: The Institutional Animal Care and Use Committees at the University of California, Davis (UC Davis; protocol #21857 and #22376) and North Carolina State University (NCSU; protocol #23-351) approved all study procedures. Study methods were designed in compliance with the ARRIVE2.0 guidelines (Percie du Sert *et al.* 2020), the Animal Welfare Act (Animal Welfare Act as Amended 2013), and the Institute for Laboratory Animal Research Guide for the Care and Use of Laboratory Animals (National Research Council (US) Committee for the Update of the Guide for the Care and Use of Laboratory Animals 2011).

### Cohort 1: animals recruited for WGS

#### Study population

As part of an effort to construct a DNA database of cats with cardiovascular disease, whole blood samples in EDTA were collected for DNA isolation from both client-owned cats seen by the Cardiology Services at the UC Davis Veterinary Medical Teaching Hospital (VMTH) and NCSU College of Veterinary Medicine (CVM) and cats within the UC Davis and NCSU communities from July 2014 to March 2023. These included HCM-affected and cardiovascularly healthy cats. All pet owners provided written informed consent prior to blood collection. A diagnosis of HCM was made in all affected cats via echocardiography and defined as end-diastolic asymmetrical or symmetrical thickening of the LVPW or IVS of ≥6 mm (Luis Fuentes *et al.* 2020). To rule out secondary causes of left ventricular hypertrophy, medical records were retrospectively scanned for documentation of a normal blood pressure (<160 mmHg) in cats of all ages, and a normal total thyroxine level (T4) in cats aged 7 years or older. Cats aged 10 years or older without any echocardiographic evidence of left ventricular wall thickening (<6 mm) or other cardiovascular diseases were labeled as controls.

#### Echocardiography

Routine echocardiograms were performed on all cats by either a board-certified cardiologist or a resident in training supervised by a board-certified cardiologist. All measurements reported in this study were obtained from the most recent full echocardiogram report recorded on either a digital off-cart workstation (Syngo Dynamic Workplace, Siemens Medical Solutions) and Studycast (Core Sound Imaging) or within the medical record. A single investigator (J.L.K.) remeasured the wall thickening of all echocardiograms available for verification of disease or control status. In 1 cat at UC Davis in which the echocardiogram was performed prior to 2015, measurements were recorded directly into the medical record, and digital imaging and commnications in medicine images were not available for reevaluation. This was because the digital off-cart workstation to store images was not yet available. At North Carolina, a total of 11 cats did not have echocardiographic measurements available for validation and were included based on diagnosis of HCM by a board-certified cardiologist.

Measurements recorded from the medical record included maximal end-diastolic thickness of the LVPW or IVS from either the 2D right parasternal long axis 4-chamber (RPLx4c) or right parasternal long axis 5-chamber (RPLx5c) views or M-mode of the right parasternal short axis view (RPSx) at the level of the papillary muscles. The overall maximal wall thickness (MWT) obtained between the LVPW and IVS was also recorded. Additional measurements recorded when available included maximal left atrial diameter (LAD) at ventricular end-systole in the RPLx4c, LAD and aortic root diameter at ventricular end-systole in the RPSx view at the level of the heart base, and end-diastolic (LVIDd) and end-systolic (LVIDs) left ventricular internal diameter on M-mode derived from the RPSx view at the level of the papillary muscles. An LA:Ao ratio (Abbott and MacLean 2006) and % of fractional shortening (%FS) were calculated as previously described (Luis Fuentes *et al.* 2020). The calculation for %FS was as follows: (LVIDd − LVIDs)/LVIDd × 100. A maximal left ventricular outflow tract velocity (LVOT_Vmax_) and left auricular flow velocity were recorded from the left apical 5-chamber (LA5c) view and left auricular view, respectively.

#### ACVIM staging

HCM-affected cats were categorized into the American College of Veterinary Internal Medicine (ACVIM) staging classification scheme previously described (Luis Fuentes *et al.* 2020). Cats were classified as having HCM ACVIM stage B2 if they had no previous evidence of CHF or arterial thromboembolic disease, and had an LA:Ao >1.8 from the RPSx view at the level of the heart base and/or a LAD (RPLx) >18 mm from the RPLx4c view at end-systole (Kittleson and Cote 2021). Subclinical HCM-affected cats with either no chamber enlargement or mild left atrial (LA) enlargement that did not meet the criteria listed immediately above were categorized as having ACVIM stage B1. HCM-affected cats that either had a diagnosis of CHF confirmed as pleural or pericardial effusion on ultrasound or pulmonary edema on thoracic radiographs or an ATE were classified as ACVIM stage C. HCM-affected cats receiving furosemide doses >6 mg/kg/day or that were nonresponsive to in-hospital therapy and euthanized or died from decompensated CHF or an ATE were categorized as ACVIM stage D. SCD did not change an affected cat’s ACVIM staging.

Where available, clinical outcomes for each cat were recorded, including whether they had experienced CHF, ATE, SCD, had evidence of spontaneous echo contrast (SEC) or a LA thrombus, and whether they died or were humanely euthanized for a cardiac or noncardiac-related cause. Cats were also evaluated for a maximal left ventricular wall thickness (WT) of ≥7 mm and echocardiographic evidence of a dynamic left ventricular outflow tract obstruction (LVOTO) at any time point during their clinical evaluation. A dynamic LVOTO was defined as 1 or a combination of the following criteria: a late-peaking flow profile on continuous wave Doppler, an LVOT_Vmax_ of >1.7 m/s measured from the LA5c view, or systolic aliasing noted within the LVOT_Vmax_ on color Doppler within either the RPLx5c or LA5c views.

#### DNA isolation and WGS

A total of 1 to 2 mL of whole blood was collected from the cephalic, saphenous, or jugular vein into EDTA blood collection tubes. DNA was either isolated from whole blood or from buffy coats after whole blood centrifugation at 2,000 × *g* for 15 min. Genomic DNA isolation was performed using commercially available kits (Gentra Puregene Bood kit, Qiagen, Hilden, Germany, and ArchivePure, 5 Prime) and by following the respective manufacturer's protocol. High-quality unfragmented DNA was selected by a combination of 1% agarose gel visualization and spectrophotometric confirmation (a 260/280 ratio of ∼1.8 and a concentration of >50 ng/μL; NanoDrop One/One, Thermofisher, Waltham, GA, USA). Samples were stored at −20 °C until ready for shipment to Theragen Bio Co., Ltd (Gyeonggi-do, Republic of Korea) for WGS. Paired-end DNA libraries were generated with a TruSeq DNA Nano library prep kit. Samples were then pooled and sequenced at ∼30× coverage on the Illumina NovaSeq6000 platform. Paired-end read lengths were either 125 or 150 bp. Reads for each sample were processed using the Whole Animal Genome Sequencing (WAGS) (Cullen and Friedenberg 2023) pipeline. Briefly, reads were mapped to the F.catus_Fca126_mat1.0 (Buckley *et al.* 2020) reference genome with BWA-MEM (Li and Durbin 2009) and variants were called with HaplotypeCaller in GATK4 (van der Auwera and Connor 2020). Variant effects were annotated with a local installation of Ensembl's variant effect predictor (VEP) (McLaren *et al.* 2016), using the associated F.catus_Fca126_mat1.0 reference gtf as a guide.

#### Genome-wide association analysis

We retained genotypes that had a minimum read depth of 4× and genotype quality greater than 20, otherwise genotypes were coded as missing. We soft filtered SNPs that were within 3 bp of an indel, indels separated by 10 or fewer bases, or met the following INFO metrics: MQ < 40, QD < 2, FS > 60, MQRankSum < −12.4, ReadPosRankSum < −8.0, SOR > 3. Genotype missingness and heterozygosity were measured with PLINK (Purcell *et al.* 2007). Prior to any analyses, we removed samples that were missing 15% or more genotypes or exhibited heterozygosity outside of 3 deviations from the mean.

We first examined for any underlying population stratification by isolating unlinked variants in PLINK (–indep-pairwise 50 5 0.2) with a minor allele frequency (MAF) >0.05 and genotype missingness <0.1 and conducted a principle component analysis. In addition, we isolated every 50th SNP from the unlinked variant list with 0 missingness, encoded heterozygous genotypes to International Union of Pure and Applied Chemistry codes, and constructed a maximum likelihood phylogenetic tree with IqTree2 allowing IqTree2 to select the best model of evolution and conducting 1,000 ultrafast bootstrap replicates (Minh *et al.* 2020).

For genome-wide association analysis (GWAS), we only tested variants that passed filters, had an MAF >0.01 with <2% missingness. We conducted GWAS for a variety of case vs control comparisons. Specifically, we independently tested the following conditions: HCM overall (109 samples); WT ≥7 mm (81); LVOTO (52); CHF (51); ATE (24); presence of SEC or a thrombus in the LA (35); all samples with either SEC or thrombus in the LA, an ATE, or both (39); SCD (3); and all samples with 1 or a combination of SCD, ATE, and CHF (57) against the control group (29). We used GEMMA 0.98.5 (Zhou and Stephens 2012) for all association testing using a linear mixed model with a relationship matrix as a random effect and included sex, age, and the first two PCs from the principle component analysis, as covariates.

#### Rare variant aggregation testing

To test if HCM-linked rare variants were localized in any specific gene, we aggregated variants by gene in the HCM–control cohort and conducted burden testing in rvtests (Zhan *et al.* 2016). We isolated PASS filtered variants with HIGH (eg frameshift, stop gained, start lost or splice variant) or MODERATE (missense or in-frame indel variants) VEP predicted impacts with an MAF <0.02 and missingness <0.1. We utilized five different models of burden tests including combined multivariate and collapsing, Zeggini, Madsen–Browning (MB), variable threshold (VT), and sequence kernel association test (SKAT) (Lee *et al.* 2014). Significance for MB, VT and SKAT were evaluated through 10,000 permutations. We conducted burden tests with the MODERATE and HIGH impact variants separately and combined. We only reported results for genes with 3 or more polymorphic variants. We also aggregated variants across progressively broader gene panels (Supplementary Table 1). The full panel included 63 genes with varying association strength with HCM in humans (Cirino *et al.* 2008; Marian 2021; Ommen *et al.* 2020). Thirty-six genes and their classifications (definitive, moderate, limited, and disputed) were drawn from clinicalgenome.org (MONDO:0005045). An additional 27 genes associated with syndromic HCM in humans: *LAMP2* (Danon disease), *GLA* (Fabry disease), *FXN* (Friedreich ataxia), *PRKAG2* (glycogen storage disease of the heart), *TTR* (hereditary transthyretin amyloidosis), *GGAP* (Pompe disease), as well as *BRAF*, *HRAS*, *KRAS*, *LZTR1*, *MAP2K1*, *MAP2K2*, *NRAS*, *PTPN11*, *RAF1*, *RASA2*, *RRAS2*, *RITI1*, *SOS1*, and *SOS2* (all of which are associated with Noonan syndrome with or without multiple lentigines, cardiofaciocutaneous syndrome, and Costello syndrome) and *ALMS1* given a hypothesized association with HCM in cats were also included.

### Cohort 2: animals recruited for RNA-sequencing

#### Study population

Cohort 2 consisted of a convenience sample of heart tissue from client-owned and purpose-bred cats humanely euthanized at UC Davis for clinical decline or outside study purposes. All cats had an echocardiogram performed immediately prior to humane euthanasia. Tissues from the LA, IVS, and LVPW were extracted and either flash-frozen in liquid nitrogen or placed in RNA later within 30 min postmortem. All samples were subsequently stored at −80 °C until ready for shipment for RNA-sequencing (RNA-Seq).

#### Echocardiography

All cats had echocardiograms performed by a board-certified cardiologist (J.L.K., J.A.S.) as described in cohort 1 immediately prior to humane euthanasia to confirm cardiovascular status of each patient. The same echocardiographic criteria outlined in cohort 1 were used to define cases and controls in cohort 2. Of those affected with HCM, disease severity was categorized based on the ACVIM staging scheme previously described with cohort 1 (Luis Fuentes *et al.* 2020; Kittleson and Cote 2021).

#### RNA-sequencing

All tissue samples available were sent to GENEWIZ (South Plainfield, NJ, USA) for RNA-Seq. Total RNA was extracted from frozen cell samples using Qiagen RNeasy Plus Universal mini kit and following the manufacturer's instructions (Qiagen, Hilden, Germany). The polyA method was used to select for mature mRNA and samples subsequently underwent quality control. RNA samples were quantified using Qubit 3.0 Fluorometer (Life Technologies, Carlsbad, CA, USA). RNA integrity was evaluated using Agilent TapeStation 4200 (Agilent Technologies, Palo Alto, CA, USA). Only samples with an RNA integrity number of >7 were converted to cDNA and processed for stranded library prep. Paired-end 150 bp cDNA libraries were sequenced on the Illumina HiSeq platform at a read depth of ∼50 million reads per sample. Illumina adapter sequences and low-quality reads were removed using Trimmomatic v 0.39 (ILLUMINACLIP:2:30:10, SLIDINGWINDOW:5:20, MINLEN:100) (Bolger *et al.* 2014). Reads were then mapped to the F.catus_Fca126_mat1.0 reference with the splicing aware STAR v 2.7.11b (Dobin *et al.* 2013). Read counts were measured using the embedded htseq-count for each annotated gene. An examination for sample stratification and outliers was conducted by generating a pairwise sample-to-sample distance matrix, a heatmap of variance stabilized read counts of the top 100 expressed genes, and a principal component analysis (PCA) in DESeq2 v1.42.0 (Love *et al.* 2014).

Differentially expressed gene (DEG) discovery was conducted with DESeq2 v1.42.0. For a gene to be tested, 10 samples had to have a minimum of 10 reads to ensure genes with robust expression were being tested. We included sex and age, as a categorical variable as either below 5 or above 5 years, as covariates. We first examined for DEGs between control and HCM cats for each tissue. We also grouped cats according to ACVIM stage, i.e. control, B1, and clinical (cats in C and D combined) and performed pairwise comparisons among each group, for each tissue. We considered genes differentially expressed if the absolute log2fold change was greater than 1 and the adjusted *P*-value was less than 0.05.

#### Gene set enrichment analysis

Gene set enrichment analysis (GSEA) for biological processes (BP), molecular function (MF), and cellular component (CC) was performed using clusterProfiler 4.10.1 (Yu *et al.* 2012) for IVS, LVPW, and LA tissues of HCM-affected cats compared to control cats. We used the human library (org.Hs.eg.db) as the GO annotation reference since the human database is among the most complete and an annotation library is lacking for the cat genome. We set the minimum and maximum gene set size to be tested between 3 and 800, respectively, and used the Benjamin–Hochberg method to adjust *P*-values for multiple testing. Similar to DEG discovery, we conducted GSEAs for control vs HCM, control vs ACVIM stage B1, control vs clinical, and B1 vs clinical. No cats in this cohort were categorized in ACVIM stage B2.

#### Statistics for clinical data

Statistical analyses for clinical comparisons were performed using commercially available software (GraphPad Prism 10.2.3, San Diego, CA, USA). All clinical variables for cohorts 1 and 2 were tested for normality using the D’Agostino–Pearson test. Descriptive statistics are reported as mean (standard deviation) and median (interquartile range) for parametric and nonparametric data, respectively. Unpaired t-tests or the nonparametric equivalent were performed between HCM-affected and control cats undergoing RNA-Seq to ensure samples were appropriately age- and weight-matched.

## Results

### Cohort 1

A total of 150 cats underwent WGS in cohort 1. However, 12 cats did not pass quality control (QC) and were removed, leaving a total of 138 samples. Of these, 109 were diagnosed with HCM and 29 were deemed cardiovascularly healthy (Supplementary Table 2). Of the total 138 cats, 18 HCM-affected cats were acquired from previous studies at the NCSU. The remaining cat samples were acquired at UC Davis. Of the HCM-affected cats, 83 were male and 26 were female. Within the control group, 12 were male and 17 were female. Breeds in the HCM-affected group included 82 Domestic Shorthairs, 6 Domestic Longhairs, 4 Maine Coons, 3 Persians, 3 Ragdolls, 3 Sphynxs, 2 Domestic Mediumhairs, 2 Siamese crosses, and 1 of each of the following: Bengal, Japanese Bobtail cross, Maine Coon cross, and Sphynx cross. Breeds included in the control group were 20 Domestic Shorthairs, 4 Domestic Longhairs and 1 each of the following: Siamese cross, Maine Coon cross, Persian, and Siberian. The median body weight for HCM-affected cats was 5.6 kg (4.8 to 6.3). The mean body weight for cardiovascularly healthy cats was 5.4 kg (1.0). The body weight for 13 HCM-affected and 9 control cats was not available for review in the medical record. The median age of diagnosis for HCM-affected cats was 5.3 years (3.6 to 9.7). The median age at which cats were deemed cardiovascularly normal on echocardiogram was 12.1 (10.8 to 13.1). The ages for 2 cats in the HCM-affected group were not available in the medical record.

Of the HCM-affected cats, a total of 81 cats had a MWT that measured ≥7 mm, 52 cats had evidence of a dynamic LVOTO, and 57 cats had 1 or a combination of severe outcomes of disease, including CHF, ATE, or SCD. Of those, 51 had CHF, 24 had ATE, and 3 experienced SCD. A total of 35 cats had evidence of SEC or a thrombus within the LA. Combining either evidence of SEC or a thrombus within the LA with cats that experienced an ATE resulted in a total of 39 cats. Total T4 measurements were not available for 22 cats and blood pressure measurements prior to initiating any medical therapy were not documented in 47 cats. All total T4 values reported were within normal reference ranges. At the time of writing, 40, 10, 26, and 28 HCM-affected cats were classified as ACVIM stage B1, B2, C, and D, respectively. A total of 5 cats were not categorized into an ACVIM stage because of the lack of medical records available.

### HCM vs control genome-wide association testing

**Fig. 1.**
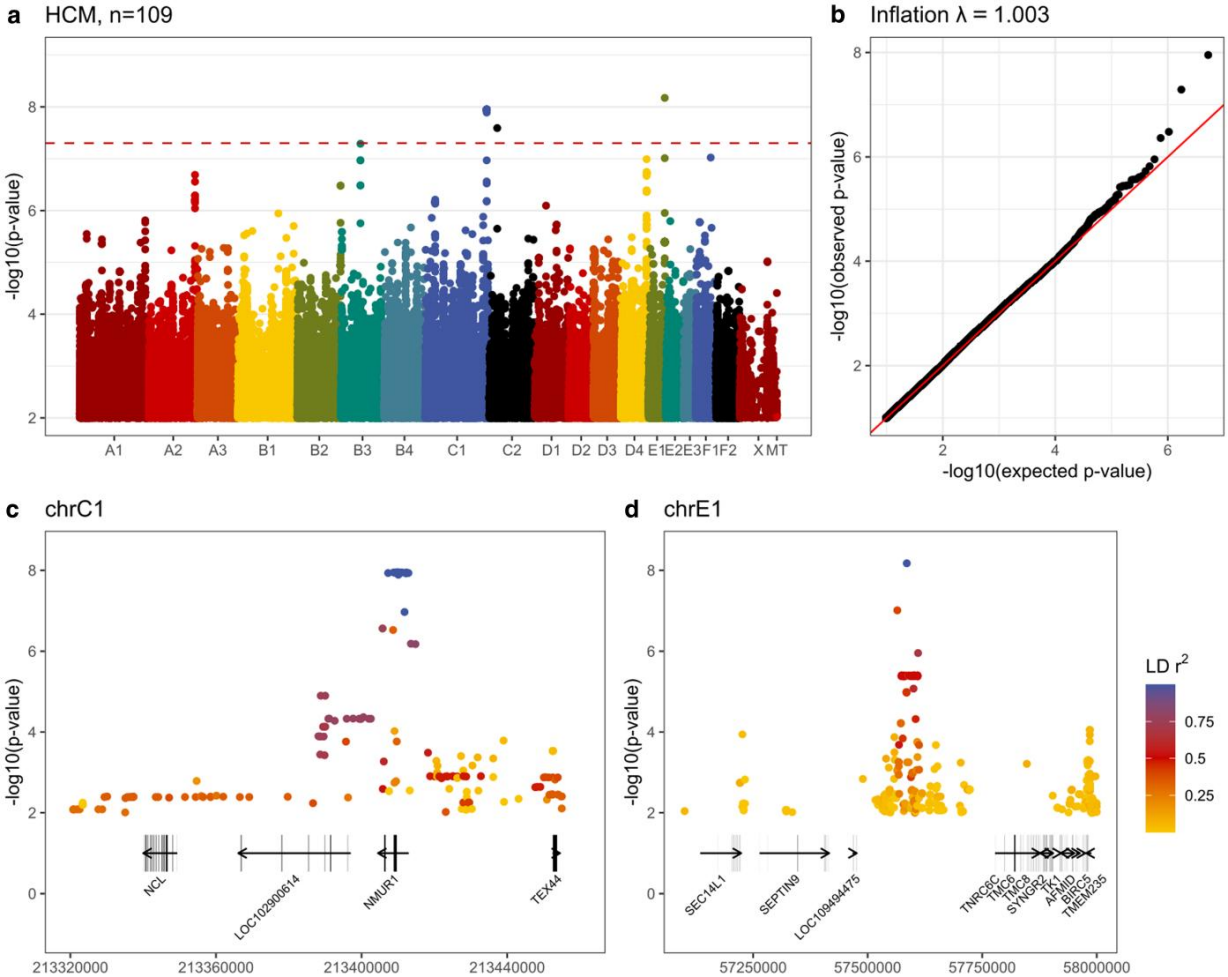
a) GWAS of HCM cases vs controls displayed as a Manhattan plot. Only variants with a *P*-value <0.01 are displayed. Genome-wide significance is shown as a dashed red line. b) QQ plot generated from a subset of 2 million unlinked variants, only *P*-values <0.1 are displayed. c) A zoomed-in plot of the peak on C1. Protein coding genes and their orientation are displayed along with coding sequence boundaries. Colors reflect the linkage disequilibrium r^2^ from the lead variant. d) Zoomed-in plot of the peak on E1.

An examination of the PCA (Supplementary Fig. 1a) and phylogenetic tree (Supplementary Fig. 1b) suggested cat breeds were largely not monophyletic, but Persian and Sphynx cats were segregated from the remaining samples. The underlying ancestry was captured by the first two PCs and was included in the GWAS as a covariate along with sex and age of the cats. To identify any common variants that could explain HCM in a majority of cats, we conducted a GWAS of variants with an MAF >0.01. About 27,842,995 SNPs passed QC and were tested, of which 281,048 had *P*-values <0.01 (Fig. 1a) and lambda calculated from unlinked variants was 1.003 (Fig. 1b). There were two windows with variants that surpassed genome-wide significance of 5 × 10^−8^. One spanned C2:213,407,301 to 213,412,872, overlapped the entire *NMUR1* (*neuromedin U receptor 1*) gene, and included 5′ untranslated region (UTR), 3′ UTR, and synonymous variants (Fig. 1c; Supplementary Table 3). The second window included 1 lead SNP at E1:57,585,439 found in intergenic space between *SEPTIN9* and *TNRC6C* (Fig. 1d). We interpret these associations with some caution as the nonreference variants were mostly present in control cats, and the sample size of the control group was relatively small. In addition, we tested for any variants that explain HCM in cats when only cats with a wall thickness ≥7 mm were included, when cats with only evidence of an LVOTO were included, or when cats of different outcomes (ie CHF, ATE, SEC or thrombus, SCD, or a combination thereof) were compared to controls (Supplementary Fig. 2). Although genome-wide lambda was generally acceptable in most comparisons, *P*-values were inflated in the tails, so we did interpret results cautiously. However, there were two consistent associations. One was on D1 in both ATE and SEC/thrombus, or both (Supplementary Fig. 2d to f). Lead variants were found in the first intron of *ACER3* (*alkaline ceramidase 3*) (Supplementary Fig. 3a). The other was on B3 in SEC, ATE, or CHF. Lead variants were found in the intron of an lncRNA (LOC109500724). Notably, LOC109500724 is approximately 1 Mb upstream of *ACTC1* (Supplementary Fig. 3b). Several variants crossed threshold and appeared significant when examining SCD, but were either intergenic, within introns or lncRNAs. Given the low sample size of 3 for SCD, *P*-values should be interpreted with caution. A list of variants with the 100 lowest *P*-values and their attributes (ie location, alleles, gene, predicted impacts, and allele frequencies) for the following comparisons are provided in Supplementary Tables 3 through 11: HCM vs control; HCM-affected cats with an MWT ≥7 mm vs control; HCM-affected cats with an LVOTO vs control; CHF vs control; ATE vs control; SEC and/or thrombus within the LA vs control; either or a combination of ATE, thrombus, and SEC vs control; SCD vs control; and either 1 or a combination of CHF, ATE, or SCD vs control.

**Fig. 2.**
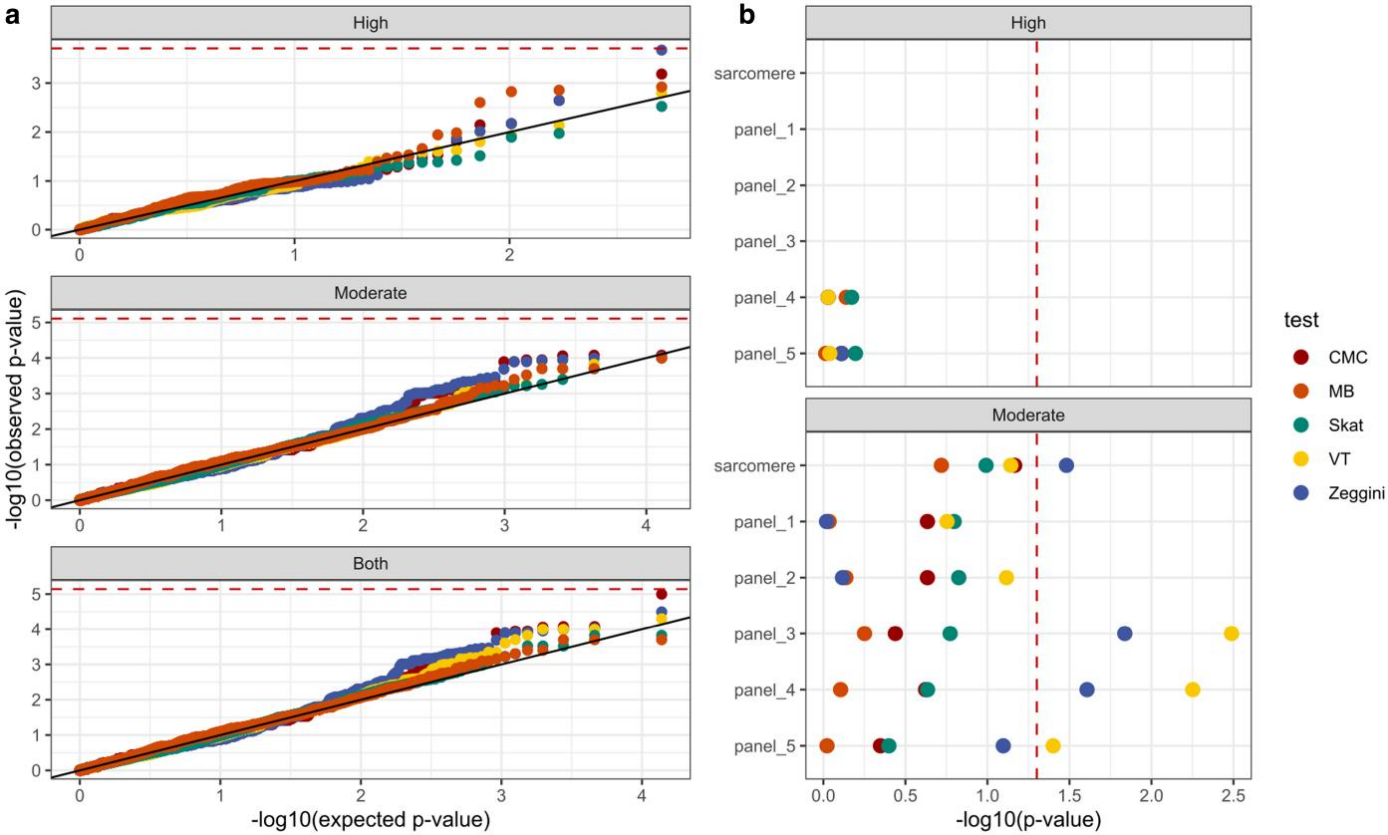
a) QQ plot for single gene association tests in HCM vs control. Colors represent different models, and the red dashed horizontal line represents the threshold based on Bonferroni correction. Panes reflect aggregation of high impact (number of tests = 255), moderate impact (number of tests = 6,419), and both high and moderate impact (number of tests = 6,904) variants combined. b) Association of high or moderate impact rare variants aggregated by panels of increasing breadth (see Supplementary Table 3). Different colors represent different statistical tests, and the vertical dashed line indicates the *P* = 0.05.

Because no significant single variants appeared convincingly associated with HCM in our affected cohort, we aggregated rare variants (MAF < 0.02) by gene and performed five models of burden testing to determine if rare variants found in HCM cats were biased in any particular gene. Unfortunately, regardless of model or impact type, no genes reached genome-wide significance (Fig. 2a). In a second approach, we aggregated variants by panels of increasing breadth, ranging from the eight core sarcomere genes to the complete panel of 63 genes that included syndromic HCM genes (Fig. 2b; Supplementary Table 1). We did this with the expectation that HCM in cats could be caused by a wide variety of rare genetic variants; therefore, a signal could be captured in broader panels. Among high impact variants, only 2 panels, a full panel of 36 genes linked to human HCM according to clinicalgenome.org (panel_4) and the entire 63 gene panel (panel_5), had enough variants to be tested; in both cases, *P*-values were close to 1 among all 5 models. Among the moderate impact variants, 2 panels, panel_3 which included up to “limited” classifications and panel_4 up to the “disputed” classification, had *P*-values <0.05 for 2 out of 5 models, but no test was significant after correction for multiple tests (*P* < 0.05/6 = 0.0083).

**Fig. 3.**
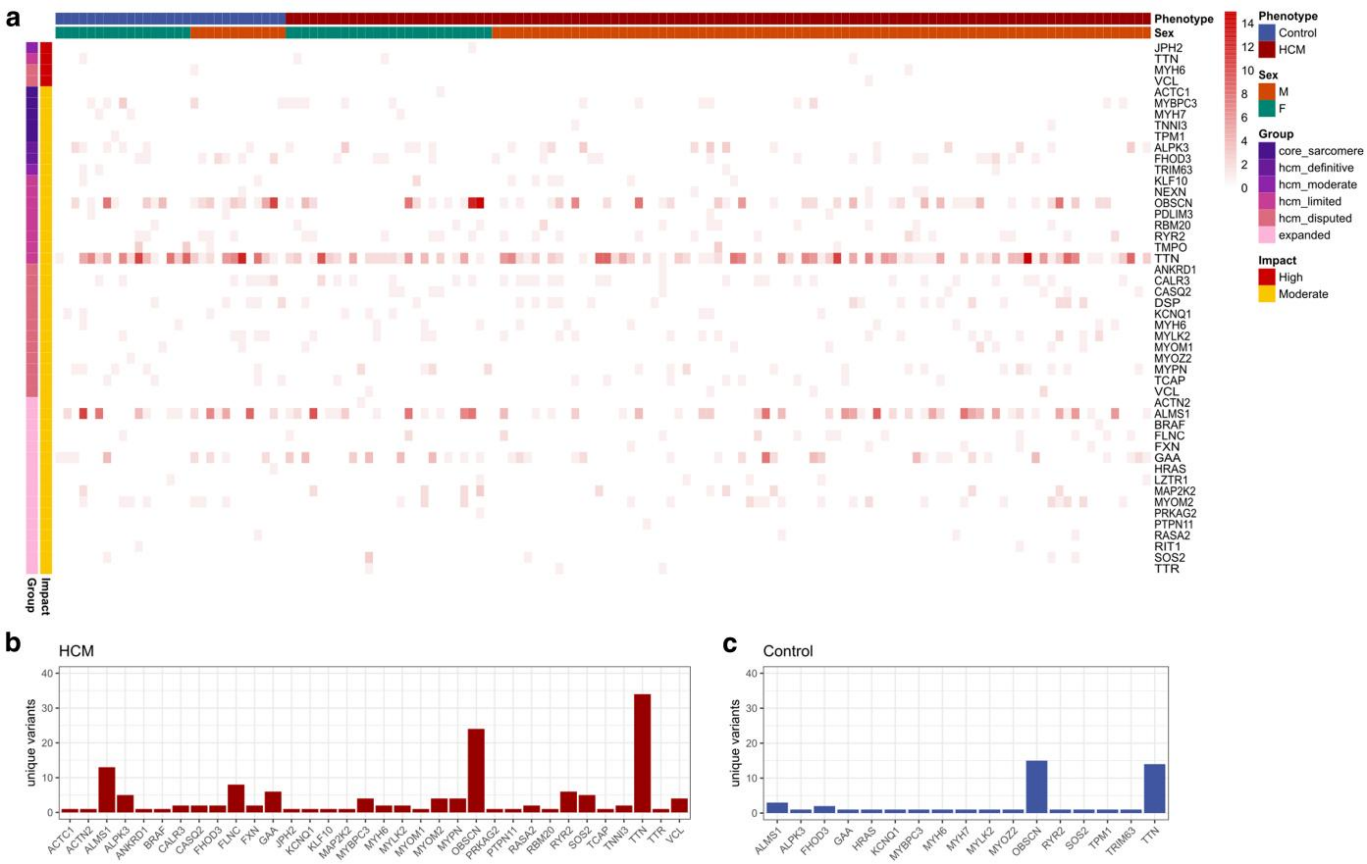
a) The number of rare high and moderate impact variants found in each sample. Counts negate whether a variant was found as a heterozygote or homozygote. Individual samples are represented along the x-axis and partitioned into their HCM or control cohorts. Color is scaled to the number of variants found in each sample, for each gene. b) Number of moderate impact variants only found in HCM cats (not in any control cats) per gene. c) Number of moderate impact variants only found in control cats (not in any HCM cats) per gene.

Because HCM is considered a heterogenous disease genotypically and phenotypically, it is possible that meaningful rare variants in genes truly associated with HCM were undetected in our statistical testing. Therefore, we visually evaluated how rare (MAF < 0.05) high or moderate impact variants were accumulating in genes in the full panel (Fig. 3a). We observed rare high impact variants within 1 control cat and 1 HCM-affected cat within *MYH6*, 3 HCM-affected cats and 1 control cat within *TTN*, 1 HCM-affected cat within *VCL*, and 1 HCM-affected cat within *JPH2*. All variants were heterozygous (Fig. 3a). In all cases, high impact variants were heterozygous.

Moderate impact variants were found in 38 genes of our full panel. The remaining 19 genes had 0 moderate variants that met our conditions including the following: *MYL2*, *MYL3*, *TNNT2*, *TNNC1*, *CSRP3*, *JPH2*, *PLN*, *FHL1*, *CAV3*, *LAMP2*, *GLA*, *KRAS*, *MAP2K1*, *NRAS*, *RAF1*, *RRAS2*, *SOS1*, *KLH24*, and *RSPKB1*. Every sample had at least 1 moderate impact variant and the highest number of rare variants was 25 found in two cats, including 1 HCM cat and 1 control. Many HCM-affected and control cats had an abundance of rare variants in large genes like *ALMS1* (∼4,000 aa), *OBSCN* (∼8,000 aa), and *TTN* (∼30,000 aa) (Fig. 3). Otherwise, rare variants appeared relatively even among HCM and control cats in the remaining genes. Notably, genes with rare variants present in only HCM-affected cats included *ACTC1*, *ACTN2*, *ANKRD1*, *BRAF*, *CASQ2*, *FXN*, *PRKAG2*, *PTPN11*, *TNNI3*, *TTR*, and *VCL* (Fig. 3a). In addition, we identified variants that were only present among HCM-affected cats in 34 genes (Fig. 3b). Excluding *ALMS1*, *OBSCN*, and *TTN*, the average number of HCM unique variants per gene was 2.6, with a maximum of 8 in *FLCN*. In contrast, variants unique to control cats were found among 17 genes (Fig. 3c), with the most observed in *OBSCN* (15) and *TTN* (14) with an average of 1.1, excluding *ALMS1*, *OBSCN*, and *TTN*. Genes that had unique variants to either group included *ALMS1*, *ALPK3*, *GAA*, *MYPC3*, *MYOM2*, *OBSCN*, *RYR2*, *SOS2*, and *TTN*.

### Cohort 2

RNA-Seq was performed in a total of 27 HCM-affected and 15 cardiovascularly normal cats. LA tissue was available in 21 HCM-affected and 13 control cats (Supplementary Table 12). LVPW tissue was available in 16 HCM-affected and 11 control cats. IVS tissue was available in 21 HCM-affected and 14 control cats. Of the HCM-affected cats, 14 were male intact, 1 was male castrated, 11 were female intact, and 1 was female spayed. Of the control cats, 10 were male intact, 4 were female intact, and 1 was female spayed. The median age of diagnosis in HCM-affected cats was 2.3 years (1.1 to 3.4). The mean ages of death were 5.2 years (2.7) and 6.1 years (2.9) in HCM-affected and cardiovascularly normal cats, respectively. The mean body weights for HCM-affected and cardiovascularly normal cats were 4.9 kg (1.1) and 4.2 kg (0.8), respectively. The body weight for 5 control cats was not available for review. There was no significant difference in age (*P* = 0.31) or body weight (*P* = 0.08) between HCM-affected and control cats.

At the time of death, there were a total of 20, 4, and 3 HCM-affected cats in ACVIM stages B1, C, and D, respectively. No cats in this cohort were classified as ACVIM stage B2. Of the HCM-affected cats, 23 were part of a purpose-bred colony of mixed breed cats harboring the variant MYBPC3:c91G>A[A31P]. Of these 23 cats, 17 were homozygous for the variant, 5 were heterozygous, and 1 was wildtype. The remaining 4 HCM-affected cats consisted of 1 purpose-bred cat from an unrelated colony and 3 client-owned cats, 2 of which died from CHF and 1 died from a combination of CHF and ATE. Of the control cats, 6 were part of the purpose-bred colony harboring the MYBPC3:c91G>A[A31P] variant. Two of these cats were wildtype and four were heterozygous, although none of these cats had phenotypic expression of their disease echocardiographically at the time of humane euthanasia. The remaining cats in the control group consisted of purpose-bred cats from an unrelated research cat colony.

An examination of a pairwise sample similarity plot (Supplementary Fig. 4a) and heatmap of the 100 most highly expressed genes (Supplementary Fig. 4b) revealed 2 outlier LA control samples that were removed prior to any analyses. There was strong differentiation of the LA tissue relative to IVS and LVPW in all examinations of the data (Supplementary Figs. 4 and 5). Furthermore, control samples and B1 samples were more similar to each other than those with ACVIM stages C and D (Supplementary Fig. 5). Therefore, we took the opportunity to examine pairwise expression differences in tissue from control, B1, and clinical (C and D) cats in addition to comparing control and HCM cohorts.

**Fig. 4.**
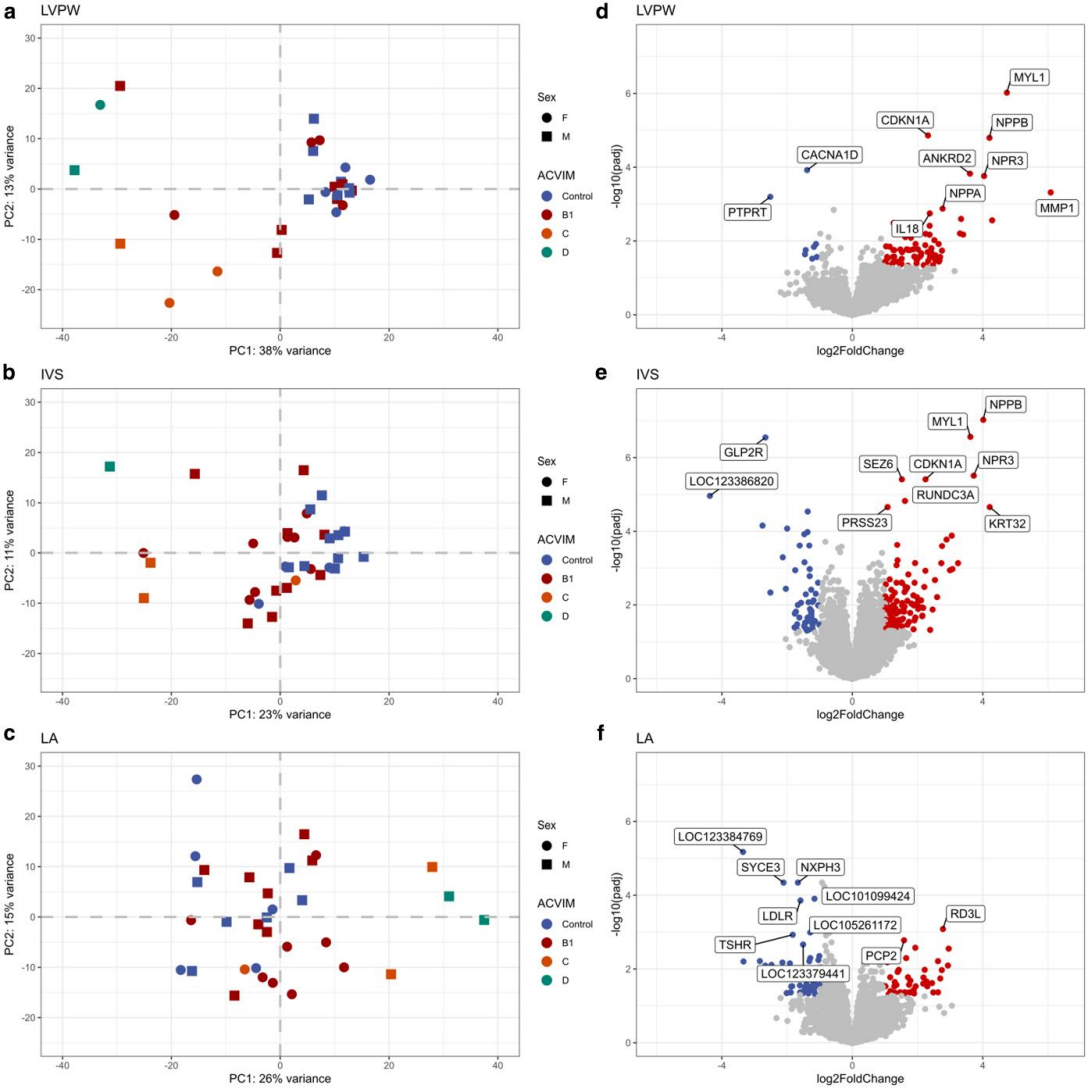
PCA plots demonstrating variance of gene expression between HCM-affected and cardiovascularly normal cats in a) LVPW, b) IVS, and c) LA tissue. Circles represent females; squares represent males; colors reflect ACVIM status. d), e), and f) show volcano plots indication log2fold expression changes between control and HCM cats. Genes significantly upregulated and downregulated in HCM cats are colored red and blue respectively for d) LVPW, e) IVS, and f) LA tissue. The top 10 protein coding DEGs according to *P*-value are shown.

A PCA plot of LVPW samples suggested that control cats and cats with ACVIM stage B1 exhibited some expression overlap compared to samples in ACVIM stages C and D (Fig. 4a), a property that was also observed in IVS and LA tissue (Fig. 4b and c). In fact, there were fewer DEGs between control and B1 samples in all tissues (Table 1). In contrast, the highest DEG counts were expectedly observed in comparisons between control and clinical samples. In general, DEGs discovered among HCM tissues largely mirrored comparisons between control and B1 cats, most likely because only 4 and 3 cats in cohort 2 were classified as ACVIM stages C and D, respectively. The top 25 upregulated and downregulated DEGs within LVPW, IVS, and LA tissue for all comparisons are shown in Supplementary Tables 13 to 24. Supplementary Tables 25 and 26 present where there is overlap between the LVPW, IVS, and LA tissue among the top 25 upregulated and downregulated DEGs between control and HCM tissues, respectively.

**Table 1.** Number of DEGs by tissue and comparison.

	Upregulated	Downregulated	Total
LVPW			
Control vs HCM	74	8	82
Control vs B1	10	2	12
Control vs clinical	1,200	539	1,739
B1 vs clinical	716	314	1,030
IVS			
Control vs HCM	115	53	168
Control vs B1	18	27	45
Control vs clinical	611	363	974
B1 vs clinical	247	138	385
LA			
Control vs HCM	45	48	93
Control vs B1	10	84	94
Control vs clinical	726	662	1,388
B1 vs clinical	507	462	969

**Fig. 5.**
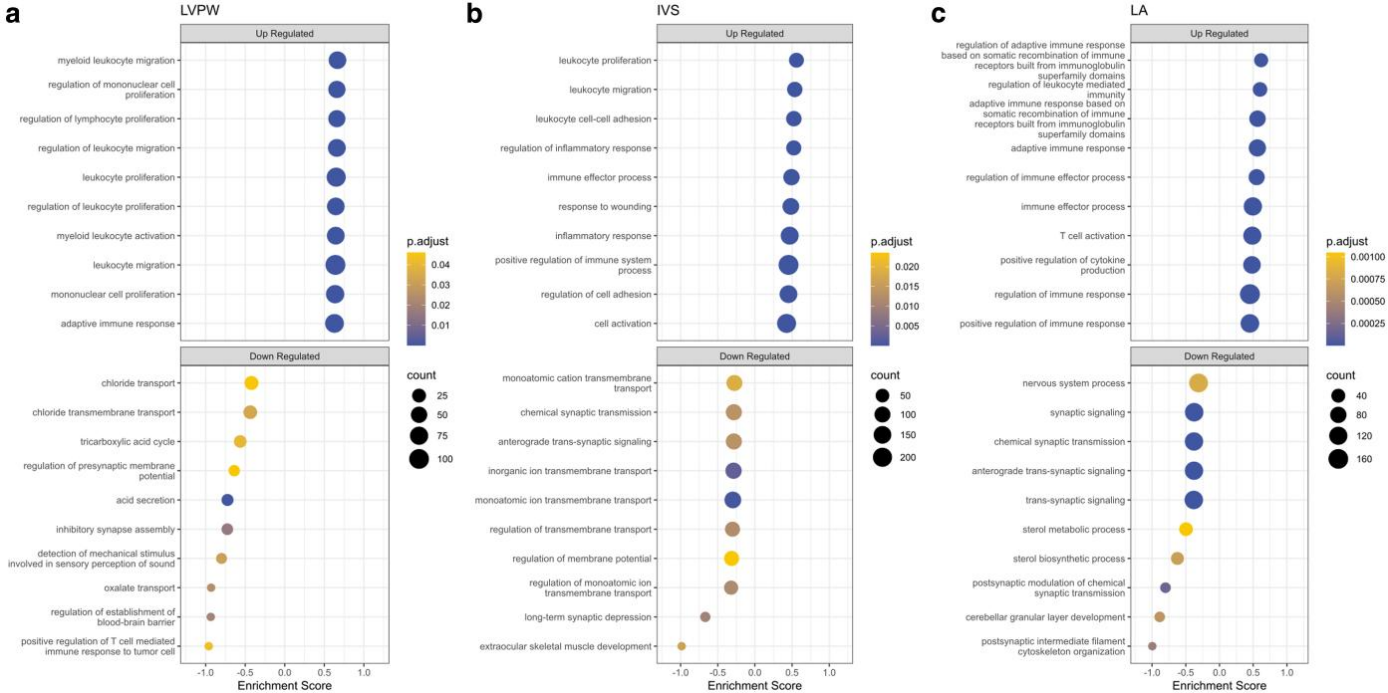
GSEA results for GO BPs in the a) LVPW, b) IVS, and c) LA tissue. The size of each point is proportional to the number of genes associated with each term. Up to the top 10 terms are listed that were up- and down-regulated in HCM cats.

Consistent upregulated BP terms regardless of comparison and tissue were immune-related processes and consistently downregulated BP terms were related to synaptic signaling (Fig. 5; Supplementary Figs. 6 to 8). Consistently, upregulated MF and CC terms indicate signaling and the extracellular environment, respectively (Supplementary Figs. 6 to 9).

## Discussion

This study offers the largest dataset of WGS and RNA-Seq in HCM-affected cats compared to cardiovascularly healthy controls. We had a relatively large sample size of HCM cats, but we were unable to convincingly yield any common risk single variants or genes with an abundance of rare variants in cohort 1 that could explain any HCM cases. One GWAS peak of interest did include variants throughout *NMUR1*, but the gene itself has not been historically linked to HCM in cats or humans. We examined whether any genes up- and downstream of *NMUR1* were differentially expressed in our RNA-Seq data indicating a regulatory impact, but no genes within a reasonable distance were differentially expressed. However, ATE and SEC associated variants in an intron of *ACER3* were relatively close to *MYO7A* (Supplementary Fig. 3a), and *MYO7A* was upregulated in the LA tissue of HCM cats (HCM vs control: log2foldChange = 1.6; adjusted *P*-value = 0.04; clinical vs control: log2foldChange = 3.5, adjusted *P*-value = 6.8 × 10^−10^), but not in IVS or LVPW tissue. The differential expression of *MYO7A* in the LA where thrombus occurs and thrombus-associated variants upstream of *MYO7A* is nonetheless intriguing and may be worthy of additional exploration. Furthermore, another GWAS peak that seemed to consistently associate with CHF and ATE/SEC was upstream of *ACTC1* (Supplementary Fig. 3b). *ACTC1* was not differentially expressed in any tissue or comparison, but *ACTC1* has been linked to HCM in humans. It is unclear if the association is biologically meaningful or what the impact is. In humans, HCM has been linked to an expansion of GAA and CGG tandem repeats in *FXN* and *DIP2B*, respectively. Although we did not get explicit hits to these regions, we did investigate if there were tandem repeats. The GAA in *FXN* was absent and the CGG repeat in *DIP2B* was only 6 units long, which is well within the normal human range. We therefore ruled out these possibilities as explanations for HCM in cats.

A recent study showed that accumulation of common genetic variants can both cause human HCM in cases in which a genetic cause is unidentified as well as increase disease severity in cases in which a genetic cause is known (Harper *et al.* 2021). We hypothesized that this may be true in the general cat population, but we lack the sample size to thoroughly investigate this possibility. We postulated that the vast heterogeneity in disease expression may have hampered our efforts to detect meaningful common variants. Given that the majority of human HCM cases can be linked to *MYH7* and *MYBPC3*, we evaluated whether rare HCM-linked variants were aggregating in any gene or group of genes associated with human HCM, but none were identified. It is interesting to note that high impact variants were truly uncommon in the investigated genes, but a few were in our HCM-affected cats, including *TTN*, *MYH6*, *VCL*, and *JPH2*. In addition, there was an abundance of rare variants of moderate impact that were unique to HCM-affected cats in *ANKRD1*, *BRAF*, *CASQ2*, *FXN*, *PRKAG2*, *PTPN11*, *TNNI3*, *TTR*, and *VCL*. Whether any of these rare variants are causative for disease is unclear but warrants future investigation. A recent study from the United Kingdom used next-generation sequencing in 23 healthy and 21 HCM-affected pedigree and domestic shorthair cats (Raffle *et al.* 2025). Screening for 18 candidate genes identified an intronic variant within the *LAMP2* gene, a 3′ UTR variant within the *TNNT2*, and 2 missense variants (1 within the *CSRP3* and 1 within a novel gene overlapping *MYH7*) that were of higher frequency in their control group compared to their HCM-affected group. This suggested that the presence of these variants may serve a protective role against the development of HCM. While our study found several variants unique to cats within the disease or control group, we were unable to classify any of these variants as disease-causing or disease-modifying. Differences in our study results may be due to a number of reasons, such as larger sample sizes or geographical divergence in study population gene pools. An alternative explanation is that external factors such as epigenetics or environment may play a larger role in HCM than previously thought (Kittleson and Cote 2021).

In cohort 2, we found several differences in gene expression between LVPW, IVS, and LA tissues of HCM-affected cats compared to controls. Among these, the *myosin light chain 1* (*MYL1*) gene and *ankyrin repeat domain 2 gene* (*ANKRD2*) were upregulated in HCM-affected IVS and LVPW tissue (Supplementary Table 25). Reports surrounding the role of *ANKRD2* gene are limited, although a recent study demonstrated that *ANKRD2* may be involved in human HCM and dilated cardiomyopathy pathways (Belgrano *et al.* 2011). Several studies demonstrate that a similar gene, *ANKRD1*, is important in human fetal heart development, and its resultant protein, cardiac ankyrin repeat protein, is upregulated in left ventricular tissue of human patients with end-stage heart failure (Zolk *et al.* 2002). Furthermore, mutations in *ANKRD1* are associated with the development of human HCM (Arimura *et al.* 2009). A study by Joshua *et al.* (2023) used RNA-Seq to evaluate gene expression in left ventricular and LA tissues of 5 cats with severe HCM and 5 healthy controls. Interestingly, we found several differences in the top 25 upregulated DEGs within our cohort compared to the results of the previous study. This may be due to differences in study design. In the previous study, only male cats were included, and all control cats were 1.5 years of age. This was significantly different from the disease group that included older cats with severe HCM phenotypes. Therefore, it is possible that some differences in gene expression reported previously were age-related or associated with heart failure rather than HCM alone. To overcome these limitations, we attempted to age-, weight-, and sex-match samples in our disease and control groups. Despite differences in methodology, both studies identified *IL18*, *MYL1*, and *ANKRD2* among the top upregulated genes within HCM-affected left ventricular tissue. Because our study analyzed LVPW and IVS tissue separately, only *MYL1* and *ANKRD2* were among the top 25 upregulated DEGs in both tissue types, and *IL18* was only among the top 25 upregulated DEGs in LVPW tissues.

Two additional genes previously identified to play an important role in the disease pathogenesis of human HCM were upregulated in IVS and LVPW tissue of our HCM-affected cats. These genes included *NPPB* and *CDKN1A* (Harper *et al.* 2021; Wehrens *et al.* 2022; Yu *et al.* 2023; Bian *et al.* 2024). In addition, *NPPA* was upregulated in LVPW tissue of our HCM-affected cats. *NPPB* and *NPPA* encode for B-type natriuretic peptide and atrial natriuretic peptide, respectively. Both hormones are important in cardiovascular homeostasis and serve as useful markers of cardiac stress (Volpe *et al.* 2014). The *CDKN1A* gene encodes for a cyclin-dependent kinase inhibitor involved in regulating the cell cycle. Studies have shown that *CDKN1A* expression is increased in human heart failure and may be involved in human coronary disease and HCM (Yu *et al.* 2023; Bian *et al.* 2024). This gene was also upregulated in left ventricular tissue in rhesus macaques with HCM (Rivas *et al.* 2024). An additional gene, *NPR3*, which encodes for the natriuretic peptide receptor 3, was upregulated in the LVPW and IVS tissue of our HCM-affected cats. *NPR3* is thought to prevent cardiomyocyte apoptosis and be an important regulator of cardiomyocyte hypertrophy (Lin *et al.* 2016). While not yet linked to human HCM, *NPR3* was upregulated in heart tissues of rat models with pressure-overload-induced cardiac hypertrophy (Christoffersen *et al.* 2006; Agrawal *et al.* 2019).

It is worth noting that there were several differences in the top 25 upregulated genes between IVS and LVPW tissues of HCM-affected cats compared to controls. Thus, the results of the current study suggest that LVPW and IVS tissues should not be used interchangeably when evaluating for differences in gene expression between diseased and control animals.

The top BP terms for upregulated DEGs within LVPW and IVS tissue were largely involved in inflammatory and immune modulatory processes. This is consistent with findings in the aforementioned study by Joshua *et al.* (2023) in which several top DEGs in the HCM-affected hearts were associated with inflammation. While HCM is largely considered an inherited disease, a study by Kitz *et al.* (2019) showed that hearts with HCM had higher transcription levels of inflammatory mediators, including IL-1, IL-6, IL-18, TNF-α, TGF-*β*, MMP-13, and TIMP-1. It was theorized that heart remodeling in HCM may be in part macrophage driven (Kitz *et al.* 2019; Fonfara *et al.* 2021). Further investigation would be required to determine to what degree upregulation of these genes impact immune modulation of the heart and whether they play a key role in HCM pathogenesis.

There were several inherent limitations to this study worth mentioning. Cohort 1 was made up of a wide variety of mixed breed and purebred cats. Combining cats of all different breeds rather than focusing on a single breed may have made it difficult to find causative genes associated with disease. However, we chose to explore HCM in a clinically relevant cohort that was more reflective of what might be seen in the general cat population. Although it is important to note that this is the largest GWAS investigation in feline HCM to date, a larger sample size may have aided in identifying any genetic variants associated with the disease. Our smaller control group made it difficult to robustly measure genetic diversity at every site and yield robust effect sizes and limited our ability to find common risk variants. Unfortunately, nonhuman variant annotations are substantially limited. With the help of SIFT, PolyPhen, CADD, LOFTEE, and gnomAD among other tools, it is easier to build robust datasets of truly rare and deleterious variants in human WGS and exome data, and these annotations are lacking for feline data. Our burden tests treated all missense variants equally, but this is not true biologically. Without a robust way to assess the impact of missense variants and select for likely deleterious variants, we were very likely to include benign missense variants that are common in the general cat population, which could yield false negatives. In addition, echocardiographic and hematological data provided were not standardized across all cats. To overcome some of these challenges, a single investigator reevaluated all echocardiograms to confirm disease phenotype or lack thereof, but a small proportion of echocardiograms were not available to review. Finally, the HCM-affected cats had a significantly higher proportion of males compared to the control group likely due to the known sex bias toward males in feline HCM (Payne *et al.* 2015; Fox *et al.* 2018). To account for this difference, sex was listed as a covariate in our GWAS.

Within cohort 2, 23 out of 27 HCM-affected cats were part of a purpose-bred colony of cats harboring the variant MYBPC3:c91G>A[A31P]. Due to their shared genetic etiology and environmental conditions, some differences in gene expression data may not be entirely reflective of the general feline population. To counter this concern, 6 out of 15 control cats were part of this same colony, 4 of which were heterozygous for MYBPC3:c91G>A[A31P] and 2 were wildtype. Although none of the control cats were phenotypically abnormal echocardiographically or grossly upon humane euthanasia, it is possible that the heterozygous genotype may have led to alterations in gene expression not yet identifiable to the naked eye. We chose to focus on transcriptomics of a clear echocardiographic phenotype in this study and believe the homogeneity of cases and controls in this cohort adds strength to its design.

Feline HCM is a genetically heterogenous disease and it is possible that many different mutations affecting small numbers of HCM-affected cats represent the true genetic etiology of this condition. Transcriptomics revealed some molecular signatures alongside pathways similarly identified in previous HCM cohorts that warrant further exploration, including those associated with inflammation. Data generated in these experiments may be used for future investigations of novel HCM biomarkers or therapeutics. Given this combinatorial data, feline HCM appears remarkably similar to that in humans where a strong cohort of affected individuals remain of unknown etiology and many possible variants may explain a small number of cases. Future studies evaluating long-range sequencing and structural variation in HCM-affected cats are warranted.

## Supplementary Material

jkaf153_Supplementary_Data

## Data Availability

All raw RNA-Seq and WGS reads are available on NCBI's short read archive under BioProjects PRJNA1194106 and PRJNA1160267. In addition, the complete WGS VCF from cohort 1 and read counts for cohort 2 are available on dryad: https://doi.org/10.5061/dryad.cjsxksnjh. Supplemental material available at G3 online.
