# Supplementary material for: Unraveling the genetics of feline hypertrophic cardiomyopathy: a multiomics study of 138 cats
Source: G3 (Bethesda). 2025 Jul 3;15(9):jkaf153. doi: 10.1093/g3journal/jkaf153 (PMC12405878; doi:10.1093/g3journal/jkaf153)
Supplement: jkaf153_Supplementary_Data [file jkaf153_supplementary_data.zip › Supplemental_Figure_Legends_G3-2025-406024.docx]

Figure S1: A) PCA of all samples in the WGS cohort. B) Unrooted phylogenetic tree of all samples. Orange colored nodes indicate bootstrap values > 80. Breed labels are the same for both panels.

Figure S2: Manhattan plots of variants when comparing HCM-affected cats in cohort 1 with A) a maximal wall thickness (WT) $\geq$7 mm, B) presence of a dynamic left ventricular outflow tract obstruction (LVOTO), C) congestive heart failure (CHF), D) arterial thromboembolism (ATE), E) presence of left atrial spontaneous echo contrast (Smoke) or thrombus, F) presence of either left atrial spontaneous echo contrast (SEC), thrombus, ATE or a combination thereof, G) any outcome (CHF, ATE, sudden cardiac death [SCD], or a combination thereof), and H) SCD compared to control cats. Genome-wide significance is shown as a dashed red line.

Figure S3. A) Zoomed in plot of the peak associated with ATE and Smoke on D1. B) Zoomed in plot of the peak on B3 associated with Smoke, ATE and CHF. In both panes, only protein-coding genes are shown to avoid crowding.

Figure S4. A) Pairwise sample-to-sample distance matrix for all tissues and samples in the RNASeq dataset. B) Variance stabilized heatmap of the top 100 expressed genes in the RNASeq dataset.

Figure S5. PCA of all samples that passed QC in the RNASeq dataset. Tissues are represented by shapes and colors reflect ACVIM status.

Figure S6. A-C) Volcano plots of pairwise DEG discovery among control, B1 and clinical samples in the LVPW. D-F) GSEA report of up to the top 10 GO MF terms up and down regulated in pairwise comparisons. G-I) GSEA report of up to the top 10 GO CC terms up and down regulated in pairwise comparisons. In all plots term enrichment is oriented toward the ‘worse’ HCM state.

Figure S7. A-C) Volcano plots of pairwise DEG discovery among control, B1 and clinical samples in the IVS. D-F) GSEA report of up to the top 10 GO MF terms up and down regulated in pairwise comparisons. G-I) GSEA report of up to the top 10 GO CC terms up and down regulated in pairwise comparisons. In all plots term enrichment is oriented toward the ‘worse’ HCM state.

Figure S8. A-C) Volcano plots of pairwise DEG discovery among control, B1 and clinical samples in the LA. D-F) GSEA report of up to the top 10 GO MF terms up and down regulated in pairwise comparisons. G-I) GSEA report of up to the top 10 GO CC terms up and down regulated in pairwise comparisons. In all plots term enrichment is oriented toward the ‘worse’ HCM state.

Figure S9. A) GSEA report of up to the top 10 GO MF terms up and down regulated in HCM cats compared to the control for all tissues. B) GSEA report of up to the top 10 GO CC terms up and down regulated in HCM cats compared to the control for all tissues.
