# Supplementary figures and images for: Unraveling the genetics of feline hypertrophic cardiomyopathy: a multiomics study of 138 cats
Source: G3 (Bethesda). 2025 Jul 3;15(9):jkaf153. doi: 10.1093/g3journal/jkaf153 (PMC12405878; doi:10.1093/g3journal/jkaf153)

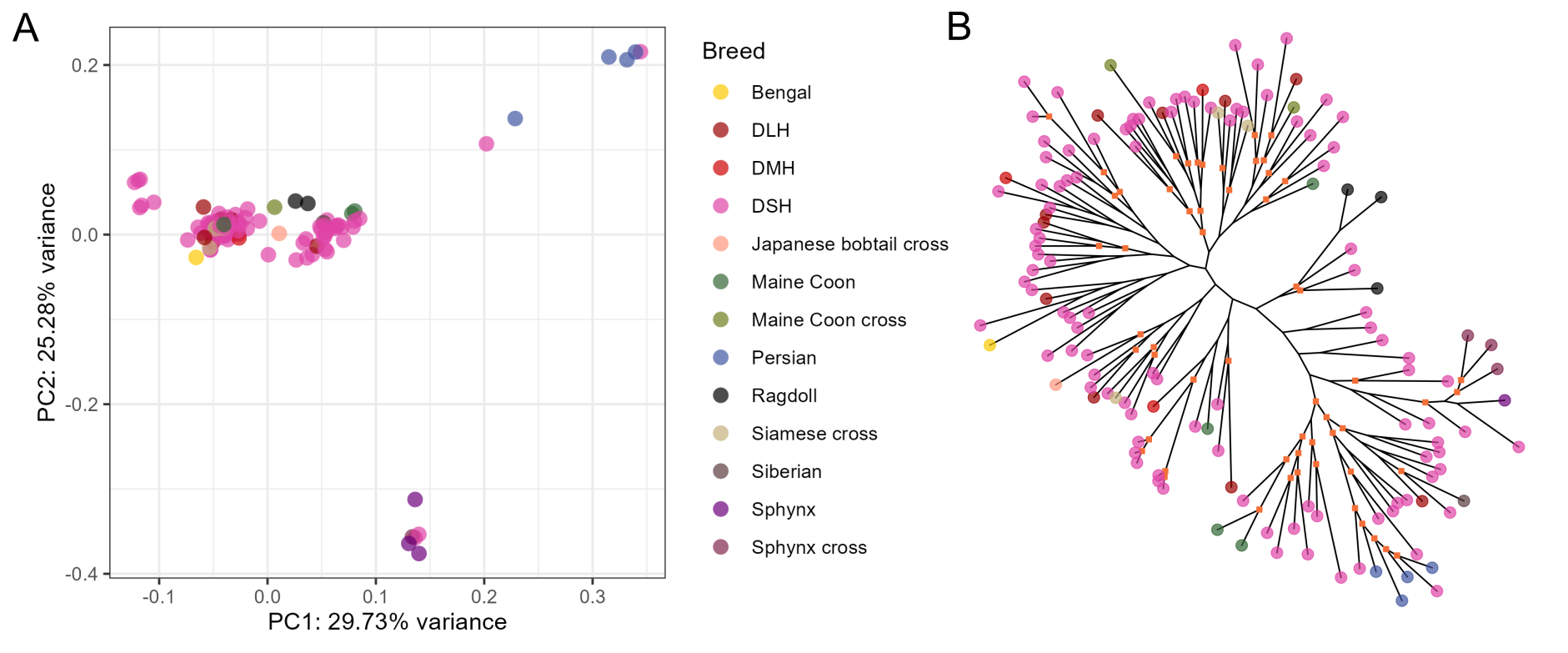

Supplement: jkaf153_Supplementary_Data [file jkaf153_supplementary_data.zip › Figure_S1_G3-2025-406024.tif]

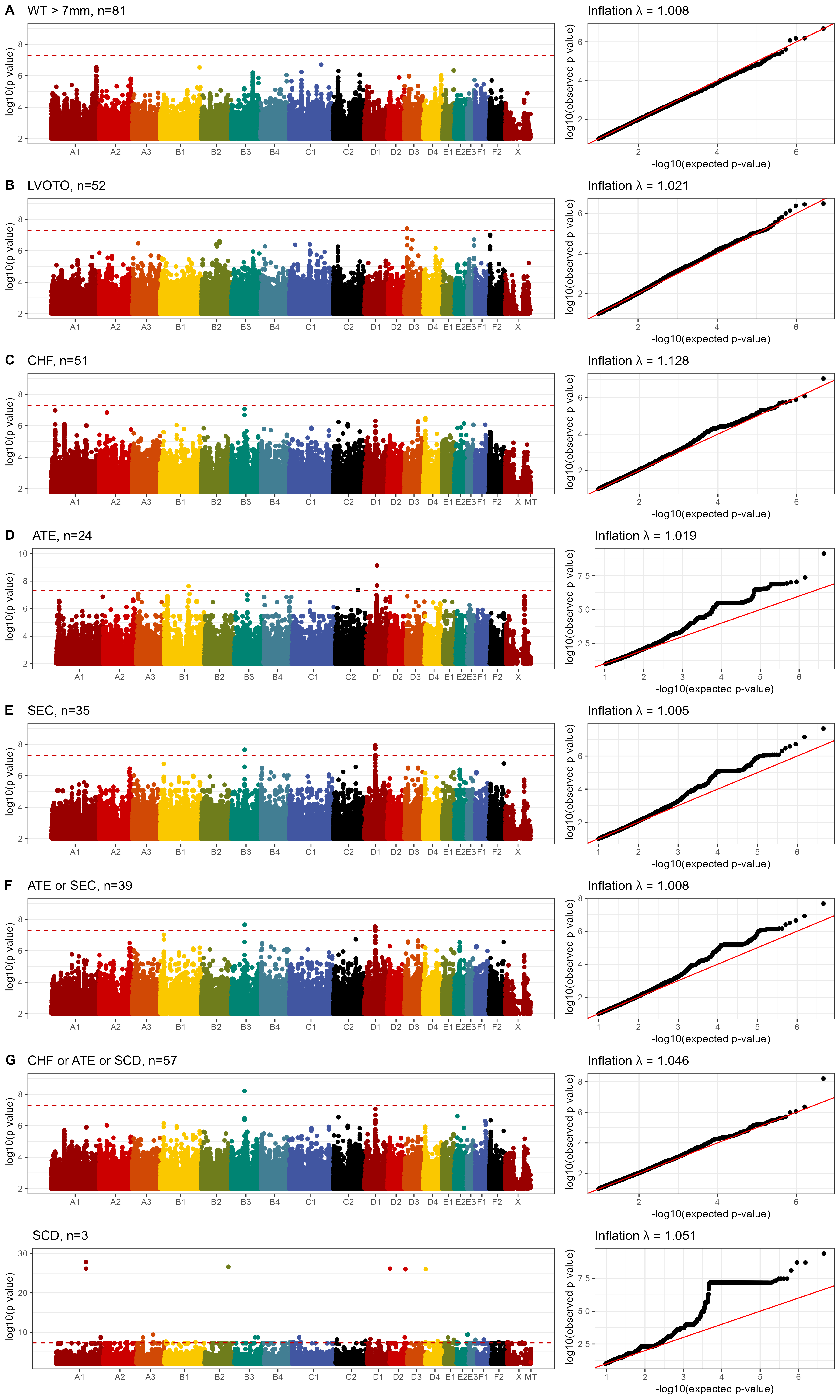

Supplement: jkaf153_Supplementary_Data [file jkaf153_supplementary_data.zip › Figure_S2_G3-2025-406024.tif]

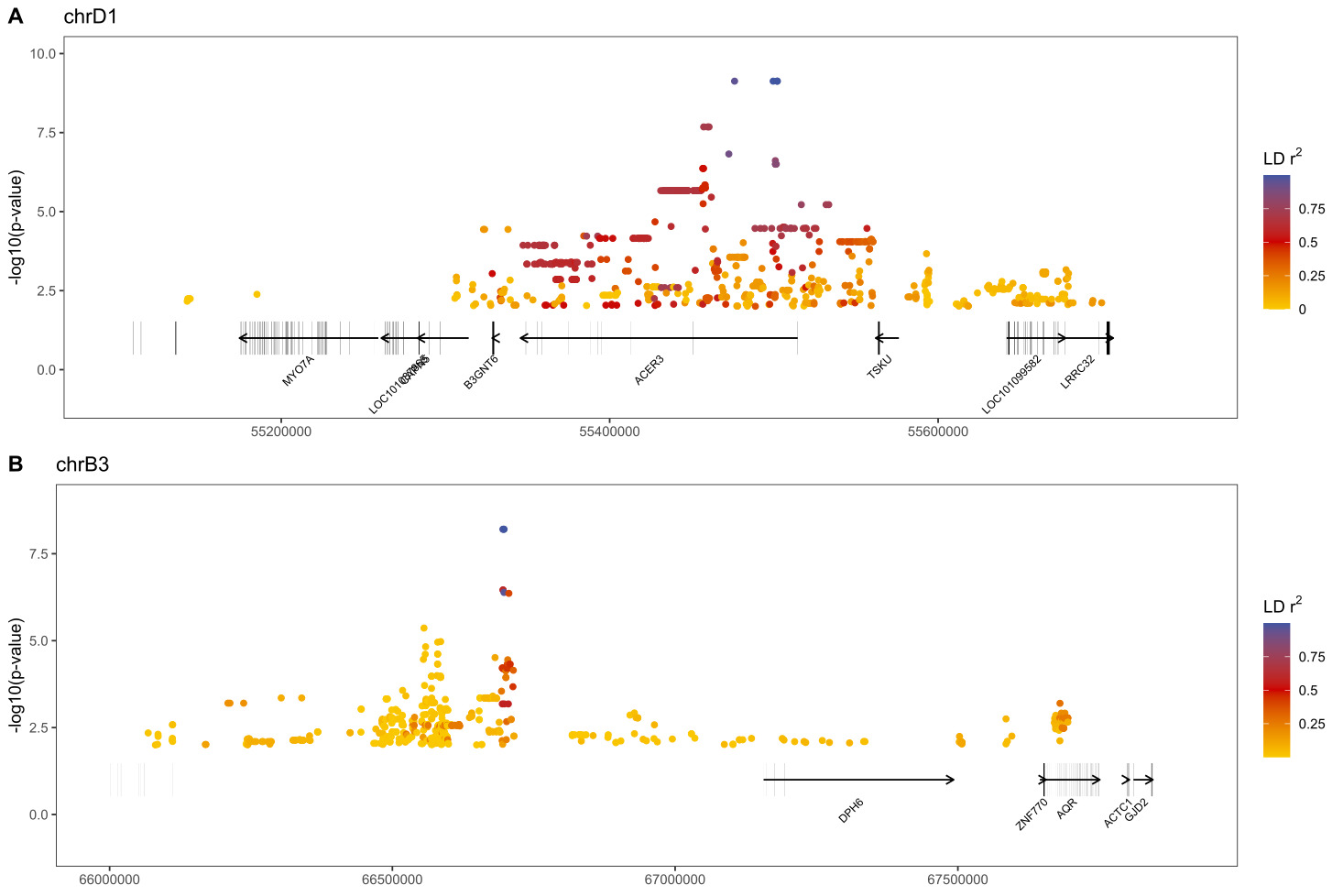

Supplement: jkaf153_Supplementary_Data [file jkaf153_supplementary_data.zip › Figure_S3_G3-2025-406024.tif]

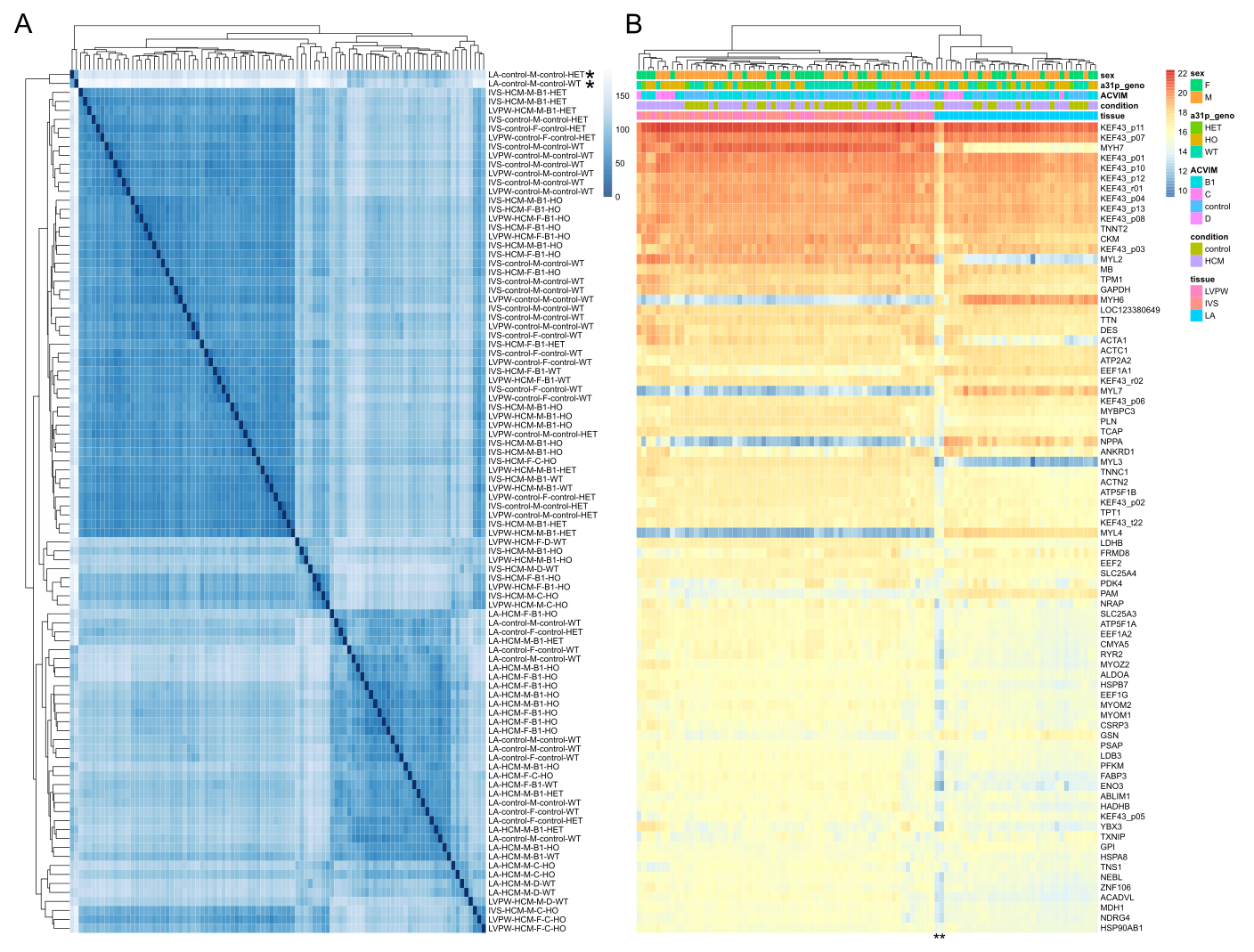

Supplement: jkaf153_Supplementary_Data [file jkaf153_supplementary_data.zip › Figure_S4_G3-2025-406024.tif]

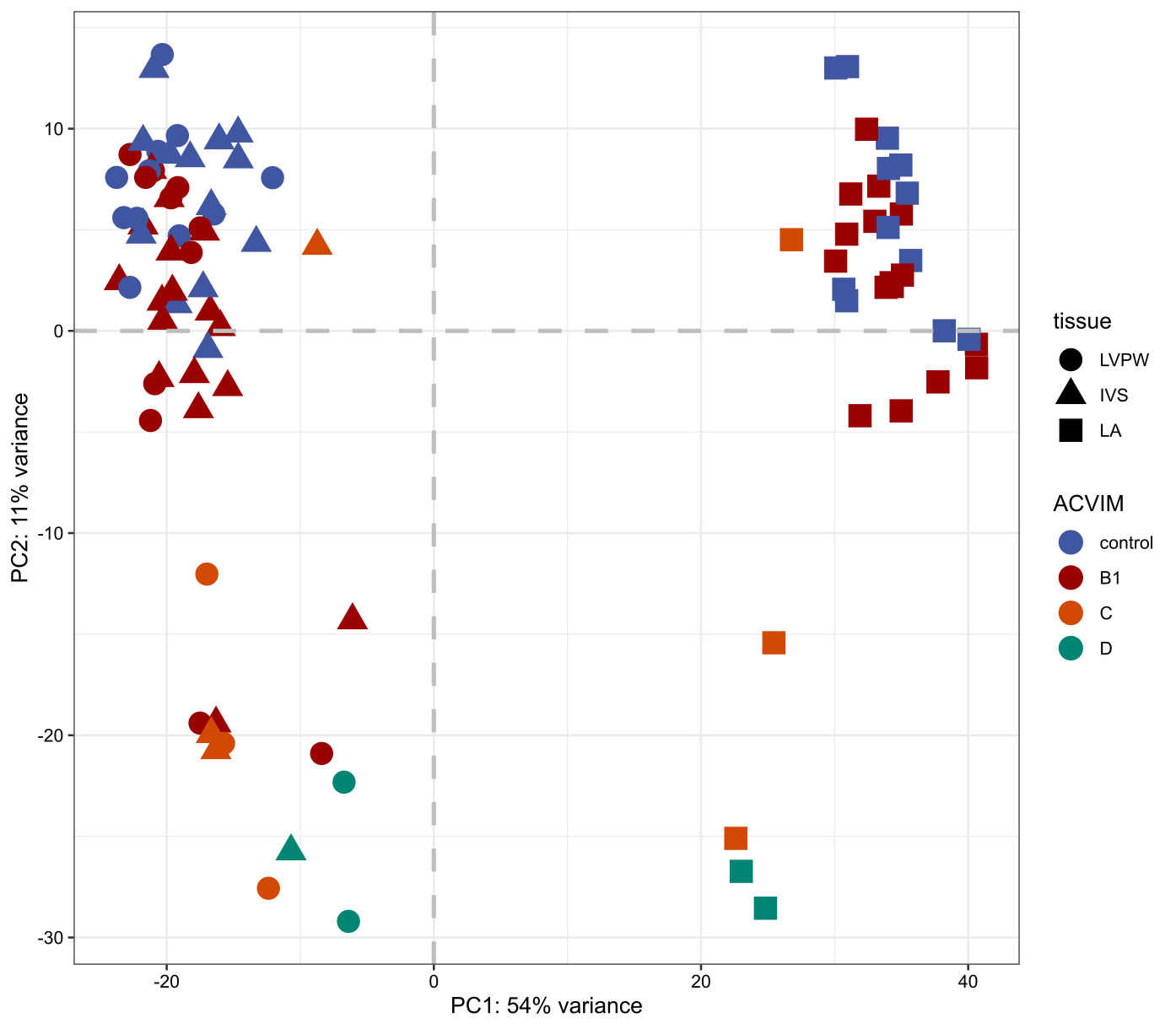

Supplement: jkaf153_Supplementary_Data [file jkaf153_supplementary_data.zip › Figure_S5_G3-2025-406024.tif]

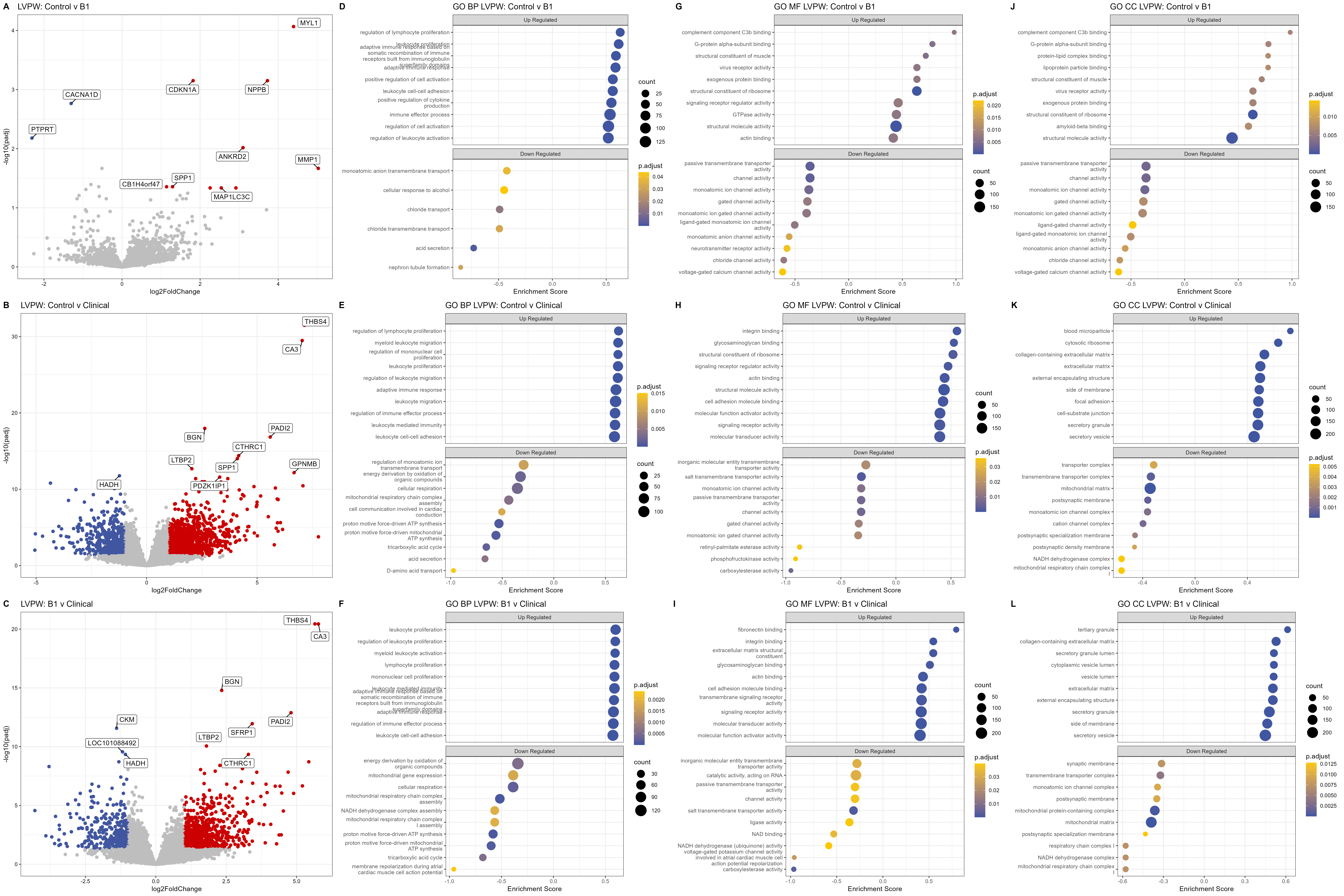

Supplement: jkaf153_Supplementary_Data [file jkaf153_supplementary_data.zip › Figure_S6_G3-2025-406024.tif]

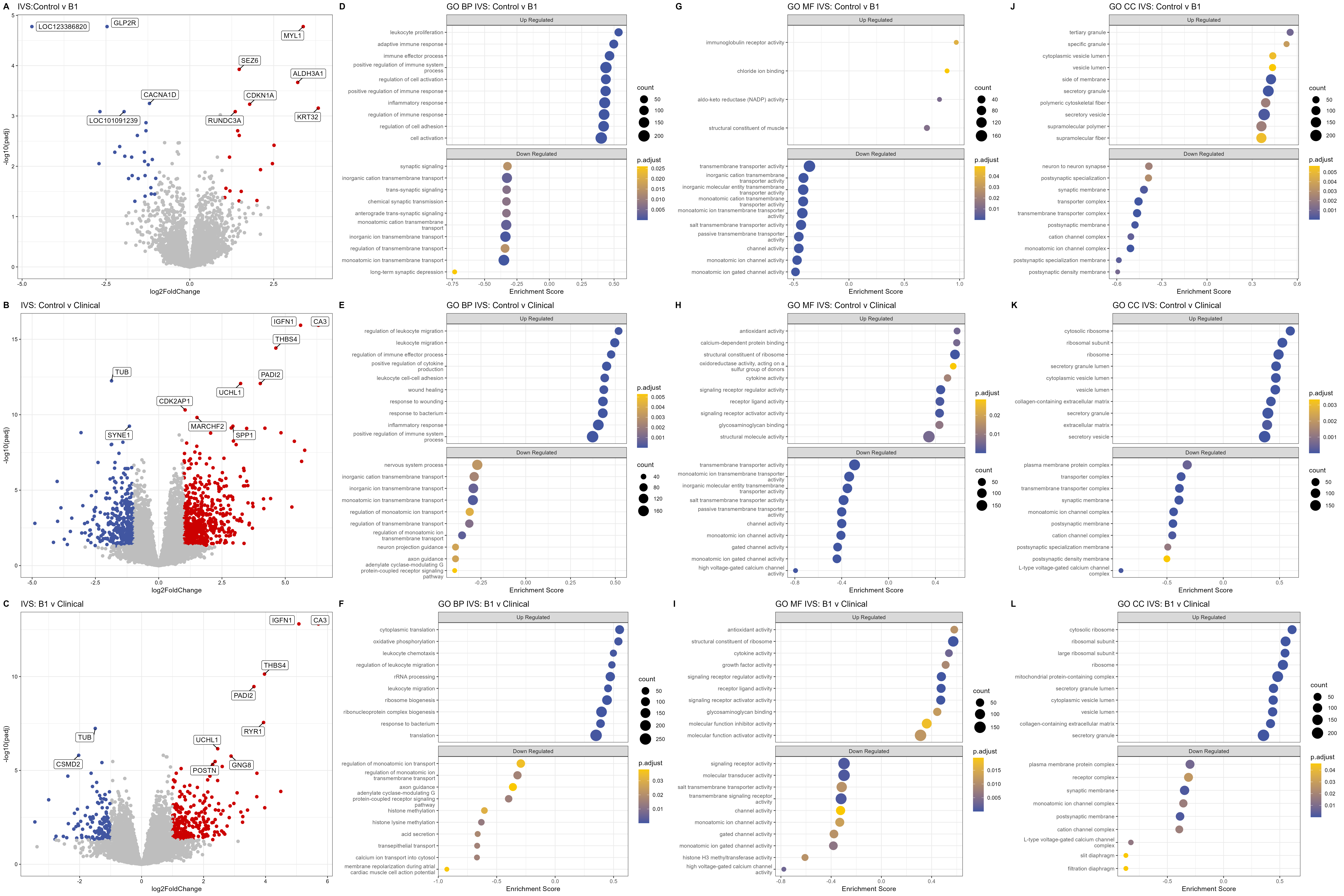

Supplement: jkaf153_Supplementary_Data [file jkaf153_supplementary_data.zip › Figure_S7_G3-2025-406024.tif]

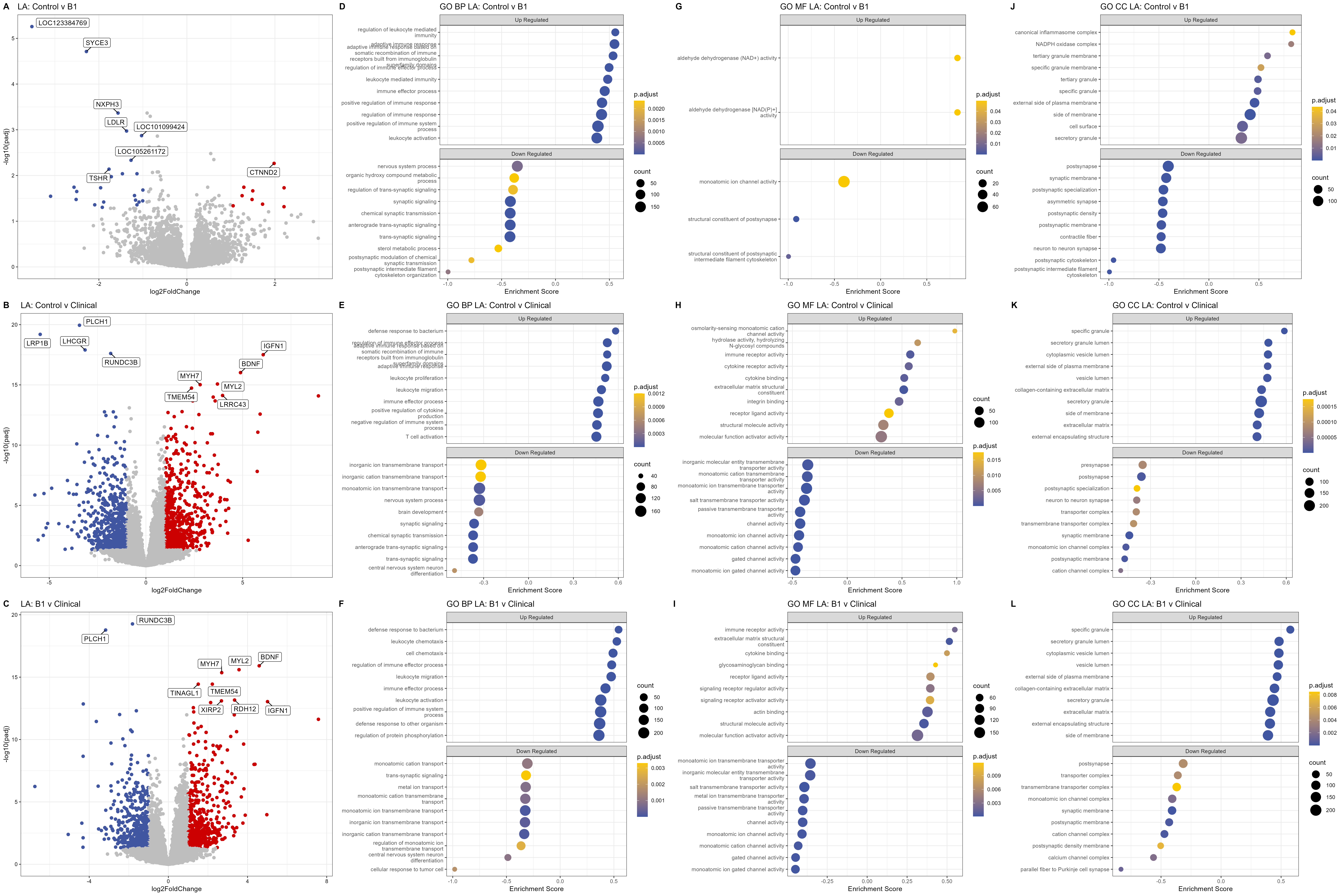

Supplement: jkaf153_Supplementary_Data [file jkaf153_supplementary_data.zip › Figure_S8_G3-2025-406024.tif]

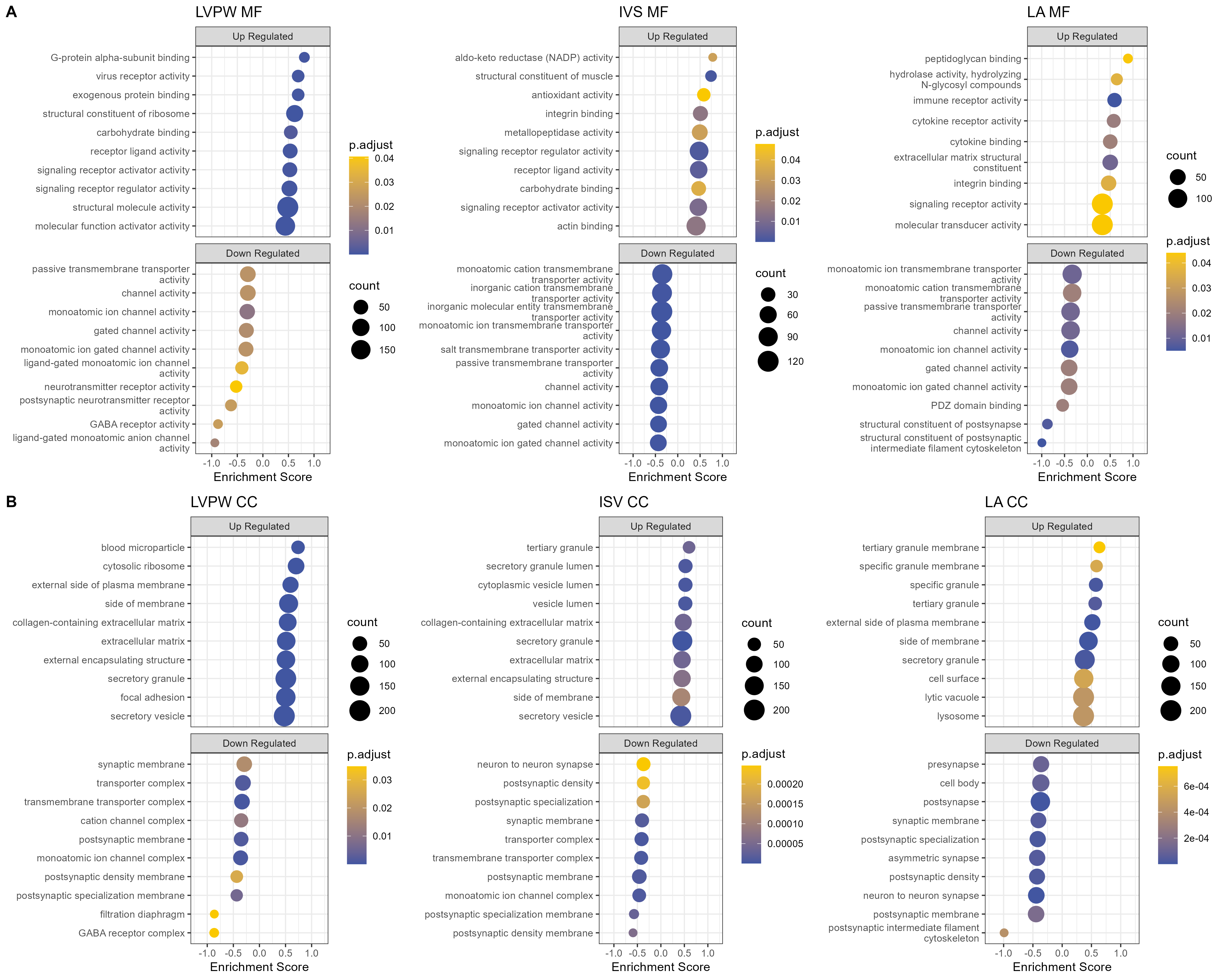

Supplement: jkaf153_Supplementary_Data [file jkaf153_supplementary_data.zip › Figure_S9_G3-2025-406024.tif]
